# GPX1-driven selenium nanoplatform reprograms MAMs-mediated organelle crosstalk to reverse inflammatory adipose expansion in thyroid eye disease

**DOI:** 10.7150/thno.117582

**Published:** 2025-08-30

**Authors:** Yao Tan, Feng Zhang, Jiamin Cao, Lemeng Feng, Bingyu Xie, Limo Gao, Xiangdong Chen, Zuyun Yan, Wei Xiong

**Affiliations:** 1 Department of Ophthalmology, The Third Xiangya Hospital, Central South University, No. 138 Tongzipo Road, Yuelu District, Changsha City 410013, Hunan Province, China.; 2 Postdoctoral Station of Clinical Medicine, The Third Xiangya Hospital, Central South University, Changsha City 410013, Hunan Province, China.; 3 The First Affiliated Hospital of Hunan University of Chinese Medicine, Hunan Ophthalmic Disease (Traditional Chinese Medicine) Clinical Research Center, Changsha City 410000, Hunan Province, China.; 4 Department of Spine Surgery, The Third Xiangya Hospital, Central South University, Changsha, Hunan, 410013, China.; 5 Postdoctoral Station of Medical Aspects of Specific Environments, the Third Xiangya Hospital, Central South University, Changsha City 410013, China.

**Keywords:** lentinan-modified selenium nanoparticles, thyroid eye disease, mitochondria-associated membranes, oxidative stress, endoplasmic reticulum stress

## Abstract

**Background:** Thyroid eye disease (TED) is a multifactorial autoimmune disorder with limited therapeutic options due to the complexity of its oxidative, metabolic, and inflammatory networks. This study aims to develop a selenium-based nanoplatform that targets mitochondria-ER interactions to reverse inflammatory adipose expansion in TED.

**Methods:** We designed a dual-responsive selenium nanoparticle (Se@LNT) modified with lentinan, capable of ROS/pH-triggered release. Human primary orbital fibroblasts, bioinformatic analysis of public datasets, and TED mouse models were used to investigate the therapeutic mechanism.

**Results:** Se@LNT undergoes intracellular metabolic conversion into selenocysteine, which enhances GPX1 activity and promotes redox balance. It exerts triple regulatory effects by stabilizing mitochondrial membranes to reduce mtDNA leakage, downregulating GRP75 to normalize MAMs contact and calcium flux, and suppressing PERK-eIF2α-ATF4 signaling to relieve ER stress. Transcriptomic profiling reveals multi-target modulation of immune-stromal interactions. *In vivo*, Se@LNT achieves orbital targeting, rapid hepatic-renal clearance, and significant reduction of adipose expansion with immune remodeling.

**Conclusions:** Se@LNT offers the first MAMs-targeted nanotherapy for TED by reprogramming organelle crosstalk, restoring metabolic-immune homeostasis, and modifying disease progression at the subcellular level.

## Introduction

Thyroid eye disease (TED) is an autoimmune disorder characterized primarily by abnormal activation of orbital fibroblasts (OFs), inflammatory adipose tissue hyperplasia, and fibrotic remodeling 1. As the most common extrathyroidal manifestation of Graves' disease (GD), TED affects up to 30% of patients and poses significant functional and psychosocial burdens 2. Current clinical treatments are limited by low efficacy and significant side effects: glucocorticoids provide only short-term inflammation control and are associated with high relapse rates and metabolic complications; surgical interventions improve appearance but cannot reverse disease progression; and targeted agents such as Teprotumumab are costly and linked to adverse effects including hyperglycemia and ototoxicity 3-5. Although selenium supplementation has shown clinical benefit by reducing oxidative stress 6, traditional formulations suffer from low bioavailability and lack of targeted delivery. These therapeutic challenges highlight the urgent need to develop safer and more effective interventions tailored to the underlying pathophysiology of TED. Research has demonstrated that the pathological adipogenic proliferation seen in TED is driven by a metabolic reprogramming mechanism cooperatively regulated by oxidative stress and endoplasmic reticulum stress (ERS) 7. Specifically, oxidative stress not only induces mitochondrial structural damage but also promotes the release of pro-inflammatory cytokines, such as TNF-α and IL-6, through activation of the NF-κB signaling pathway 8, 9. Notably, excessive accumulation of reactive oxygen species (ROS) impairs the unfolded protein response (UPR) by oxidatively modifying endoplasmic reticulum chaperone proteins, such as BiP, thereby diminishing their protective functions and ultimately sustaining ERS activation 10. Persistent ERS further drives the differentiation of OFs into adipocytes by upregulating adipogenic transcription factors PPARG and CEBPA through the PERK/eIF2α/ATF4 signaling axis, and enhancing the expression of key lipid droplet synthesis enzymes FABP4 and PLIN1 11, 12. However, current therapeutic interventions have substantial limitations in effectively controlling disease progression, emphasizing an urgent need to develop novel targeted therapies rooted deeply in these pathological mechanisms.

Given the intricate pathogenesis of TED, therapeutic strategies that solely target ROS or ERS have often proven insufficient in achieving satisfactory clinical outcomes. Emerging studies have identified mitochondria-associated endoplasmic reticulum membranes (MAMs) as a critical interface orchestrating mitochondrial-ER crosstalk, particularly through integrated regulation of calcium homeostasis, lipid metabolism, and mitochondrial quality control 10, 13, 14. Nevertheless, the precise role of MAMs in the onset and progression of TED remains largely unexplored. Mechanistically, MAMs contribute to TED pathogenesis by: 1) mediating calcium homeostasis disruption, which induces mitochondrial Ca^2+^ overload, subsequently activating NADPH oxidase and exacerbating ROS generation 15; 2) dysregulating lipid metabolism (such as impaired cholesterol esterification and fatty acid oxidation), thus promoting excessive adipose tissue proliferation 16; and 3) amplifying pro-inflammatory signaling, notably through NLRP3 inflammasome activation and mitochondrial DNA (mtDNA) release, thereby elevating levels of inflammatory cytokines including IL-1β 17. Based on these insights, developing therapeutic strategies that concurrently modulate calcium signaling, rectify lipid metabolic disturbances, and restore mitochondrial integrity by targeting MAMs represents a promising, multi-pathway approach to more effectively mitigate the pathological progression of TED.

Recent studies have revealed a significant pathological link between MAMs dysfunction and abnormal selenium metabolism 18. Selenium, a crucial trace element in thyroid metabolism, mediates thyroid hormone homeostasis, oxidative stress clearance, and immune balance via 25 distinct selenoproteins, including glutathione peroxidases (GPXs) 19-21. Among these, selenium-dependent GPX enzymes, featuring selenocysteine (SeCys_2_) at their catalytic cores, efficiently eliminate ROS, thereby mitigating mitochondrial oxidative stress and ERS, and consequently modulating aberrant MAM anchoring 17, 22-24. Accumulating evidence demonstrates that selenium supplementation substantially protects OFs from oxidative injury and attenuates inflammatory cytokine secretion by inhibiting the NF-κB signaling pathway 25, 26. However, conventional selenium preparations are hindered by notable limitations, including singular chemical valence states, intricate metabolic pathways, poor bioavailability, non-specific tissue accumulation, and narrow therapeutic windows 20, 27. Additionally, they lack stimuli-responsive drug release capabilities tailored to pathological microenvironments, hindering precise regulation within target lesions. In contrast, selenium nanoparticles, owing to their high surface-area-to-volume ratio and customizable ROS-responsive features, exhibit distinct therapeutic advantages 27, 28. Notably, Zou et al. demonstrated that lentinan (LNT)-functionalized selenium nanoparticles (SeNPs) are metabolically converted into SeCys_2_, thereby activating GPX1 and suppressing osteoclast differentiation 29. This finding provides a mechanistic foundation for our study, which builds upon and extends this strategy by designing Se@LNT, a dual-responsive nanoplatform targeting GPX1/MAM axis to reverse inflammatory adipogenesis in TED.

**Scheme 1:**
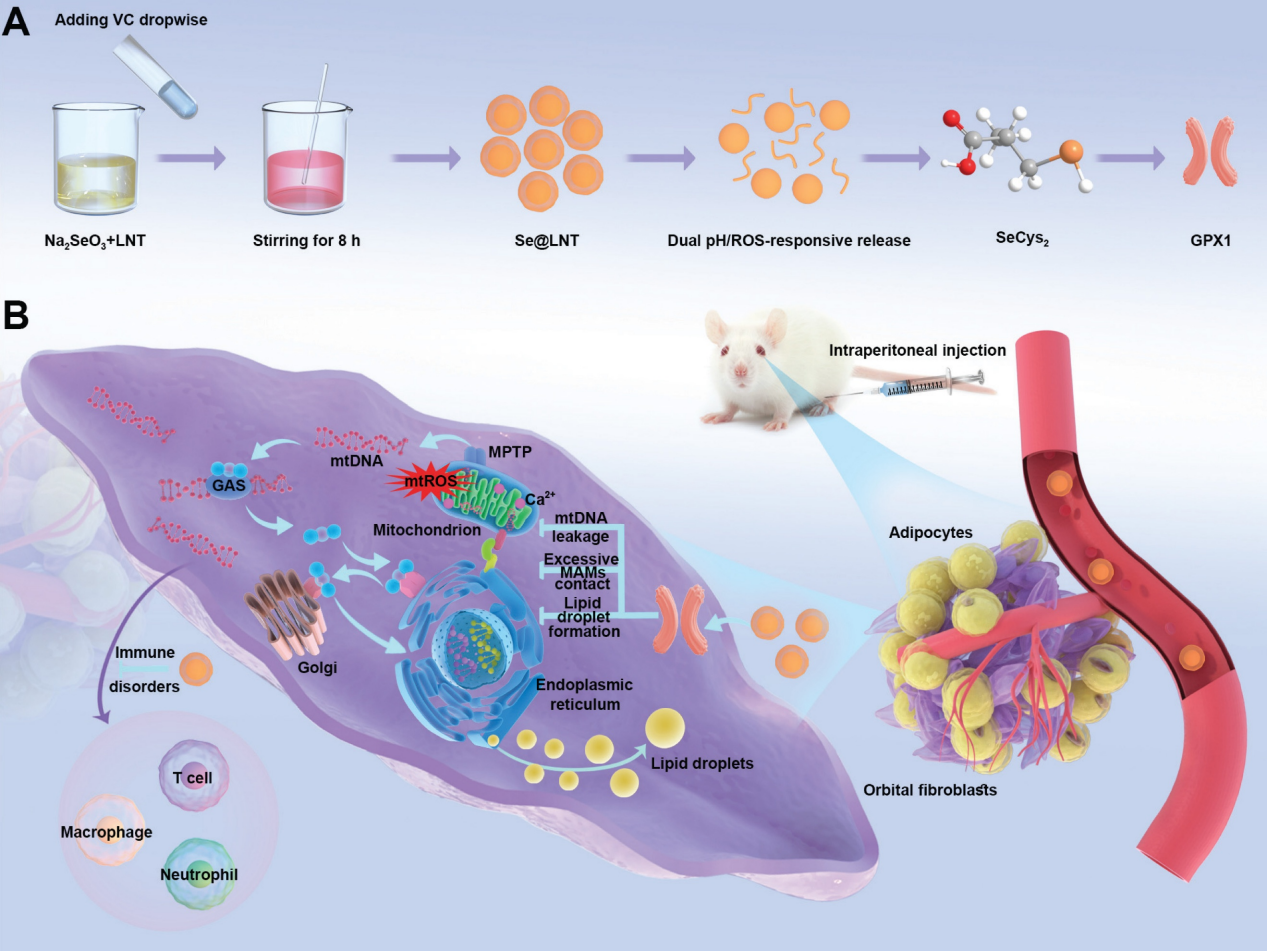
Synthesis and mechanism of action of lentinan-modified selenium nanoparticles (Se@LNT). (A) Schematic illustration of Se@LNT synthesis. (B) Molecular mechanism underlying the therapeutic effect of Se@LNT in TED-associated inflammatory adipogenesis through GPX1-mediated modulation of calcium homeostasis at MAMs.

Building upon the aforementioned research context, this study constructs a lentinan-modified selenium nanoparticles (Se@LNT) platform tailored specifically to the intricate pathological microenvironment of TED, following a comprehensive "structural adaptability-temporal responsiveness-mechanistic synergy" triadic strategy. Surface modification with LNT, abundant in hydroxyl functional groups, establishes stable interfacial interactions with selenium nanoparticles, markedly enhancing their dispersibility, stability, and permeation into orbital adipose tissue. This advanced system achieves significant breakthroughs by: (1) employing a dual-modal responsive drug release mechanism through the cooperative interplay of Se-O coordination bonds and ROS-sensitive moieties; (2) establishing a sophisticated metabolic-immune crosstalk regulatory network via the integration of GPX protein-mediated antioxidant repair, calcium homeostasis modulation, and immunoregulatory processes (Scheme 1). The physicochemical properties and microenvironment-responsive release behaviors of Se@LNT were systematically characterized, and its therapeutic efficacy and precise modulatory mechanisms in TED were rigorously validated through multidimensional experiments including primary OF adipogenic differentiation assays, ultrastructural analyses, and TED animal models. Collectively, this research not only facilitates a functional transition of selenium nanoparticles from conventional antioxidants to organelle-interface modulators but also offers a promising therapeutic strategy featuring multi-target synergy for the clinical management of TED.

## Results and Discussion

### Dysregulated selenium metabolism in TED

**Figure 1:**
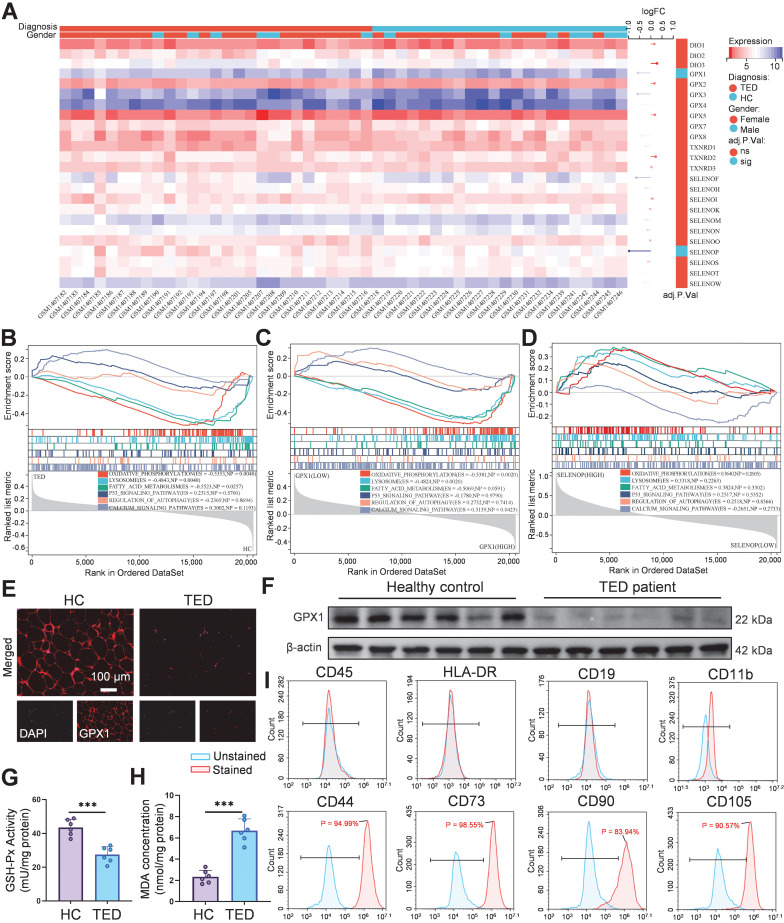
Selenium metabolism characteristics and GPX1 expression in orbital tissues of TED patients. (A) Heatmap of 24 screened selenium metabolism-related genes. (B) Pathway enrichment analysis of differentially expressed genes in the TED group. (C) GSEA pathway analysis based on median GPX1 expression. (D) GSEA pathway analysis based on median SELENOP expression. (E) Immunofluorescence staining of GPX1 in orbital adipose tissue. (scale bar: 100 μm) (F) Western blot analysis of GPX1 protein expression in orbital adipose tissues (*n* = 6). (G) GSH-Px activity and (H) MDA levels in orbital adipose tissues; ***p < 0.001 (mean ± SE, *n* = 6). (I) Flow cytometric phenotyping of primary orbital fibroblasts. HC, Healthy control.

Accumulating clinical and experimental evidence suggests that selenium metabolism is dysregulated in GD and TED. Several studies report that serum selenium concentrations in GD patients are significantly lower than those in healthy individuals, particularly in regions with marginal selenium intake such as Europe. In TED, selenium deficiency appears even more pronounced and is inversely correlated with TRAb titers, indicating a potential link between oxidative stress, selenium status, and disease severity 9, 30. Excessive TRAb activity leads to overactivation of the mitochondrial electron transport chain, triggering excessive superoxide generation, mitochondrial dysfunction, and ROS accumulation 31, suggesting a pivotal role for selenium metabolism dysregulation in TED progression. Previous randomized trials have demonstrated that selenium supplementation improves clinical outcomes only in individuals with baseline selenium levels below 122 μg/L, reinforcing the clinical relevance of selenium deficiency in TED pathogenesis 6. In addition, orbital fibroblasts from TED patients show a distinct response to oxidative stress and selenium treatment compared with controls. *In vitro* studies confirmed that selenium attenuates H_2_O_2_-induced oxidative damage, proliferation, and hyaluronic acid release specifically in TED-derived fibroblasts 25. To elucidate the molecular role of selenium in TED, we integrated bioinformatics analysis with molecular validation. Transcriptomic data from orbital tissues of TED patients (*n* = 27) and healthy controls (*n* = 22) were obtained from the GEO dataset GSE58331 32. Among 24 selenium metabolism-related genes screened, GPX1 (logFC = -0.570, adjusted *p* = 0.048) and SELENOP (logFC = -0.964, adjusted *p* = 0.016) were significantly downregulated (Figure 1A). GPX1, a key antioxidant enzyme, maintains redox homeostasis by scavenging hydrogen peroxide (H_2_O_2_) and lipid hydroperoxides 33. GSEA revealed significant suppression of oxidative phosphorylation (ES = -0.535, *p* = 0.004), lysosome (ES = -0.484, *p* = 0.004), and fatty acid metabolism (ES = -0.552, *p* = 0.026) pathways in TED tissues (Figure 1B). Inhibition of oxidative phosphorylation reflects mitochondrial dysfunction, while disordered fatty acid metabolism may contribute to lipid accumulation, consistent with the adipogenic phenotype of TED 7, 31. Stratification by median GPX1 expression further demonstrated significant inhibition of oxidative phosphorylation (ES = -0.538, *p* = 0.002) and lysosomal pathways (ES = -0.482, *p* = 0.002), along with activation of calcium signaling (ES = 0.314, *p* = 0.042) in the low-GPX1 group (Figure 1C), highlighting GPX1 as a potential therapeutic target for TED. No significant pathway enrichment was observed upon stratification by SELENOP expression (Figure 1D). Immunofluorescence staining and Western blot analysis confirmed that GPX1 protein levels were markedly higher in control tissues than in TED samples (Figure 1E-F; Figure S1). Additionally, reduced GSH-Px activity (Figure 1G), decreased GSH/GSSG ratio (Figure S2), and elevated malondialdehyde (MDA) levels (Figure 1H) were observed in TED patients, collectively indicating heightened oxidative stress. These findings align with previous clinical studies consistently reporting significantly increased ROS generation and oxidative stress markers in TED patients. For example, Hondur et al. demonstrated elevated plasma lipid peroxidation markers and impaired antioxidant capacity in TED patients relative to healthy individuals 34. Similarly, Tsai et al. reported increased oxidative DNA damage and heightened oxidative stress in TED orbital tissues 35. The impaired GPX1 activity observed here likely exacerbates ROS accumulation by limiting effective peroxide detoxification, thereby perpetuating oxidative damage and inflammation in TED.

Subsequently, OFs were isolated from both TED patients and healthy controls, and characterized by flow cytometry. The isolated cells exhibited positive expression of CD44, CD73, CD90, and CD105, consistent with a mesenchymal stem cell (MSC)-like phenotype, and negative expression of hematopoietic (CD45), T lymphocyte (HLA-DR), B lymphocyte (CD19), and macrophage (CD11b) markers, confirming high cellular purity (Figure 1I; Figure S3) 36. This surface marker profile aligns with the International Society for Cellular Therapy criteria for MSC identification, and has been widely used to characterize primary OFs 37. To assess baseline mitochondrial function, JC-1 and MitoSOX Red staining were performed. Both healthy and TED-derived OFs showed relatively preserved mitochondrial membrane potential (MMP) and low mtROS levels at baseline, likely due to the absence of inflammatory stressors *in vitro*. However, upon oxidative stimulation with H_2_O_2_ (200 μM for 24 h), TED-derived OFs exhibited a significantly greater MMP collapse and mtROS increase than healthy controls (Figure S4-S5), indicating heightened oxidative susceptibility. These findings align with the known vulnerability of TED tissues to redox imbalance and validate our choice of TED-derived OFs for subsequent experiments 7, 38, 39. Further analysis confirmed that GPX1 expression was significantly downregulated in H_2_O_2_-treated TED-derived OFs (Figure S6-S7), consistent with the patterns observed in patient tissues. This redox-sensitive phenotype provides a disease-relevant model for mechanistic studies and Se@LNT-based therapeutic evaluation.

### Synthesis and characterization of Se@LNT

**Figure 2:**
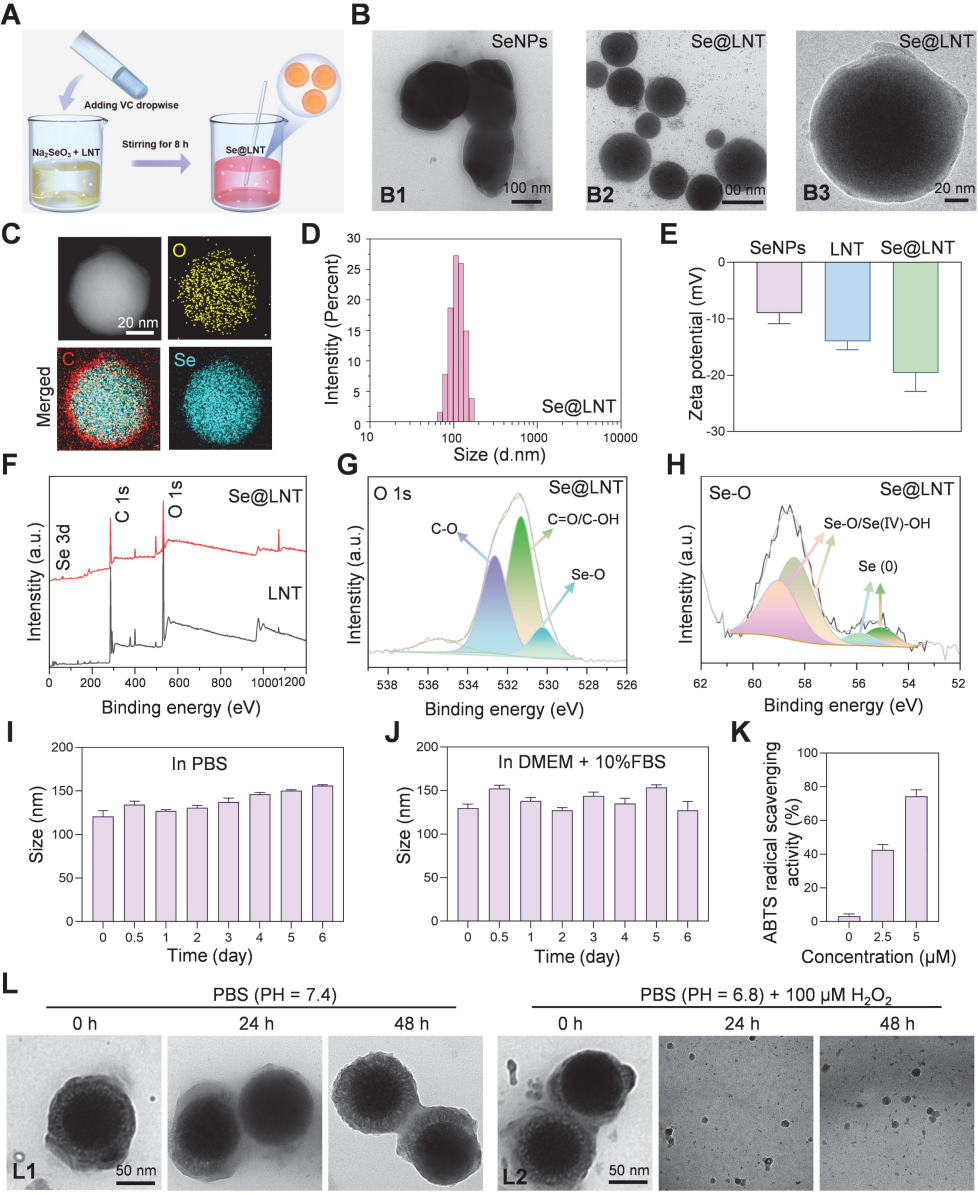
Synthesis and characterization of Se@LNT. (A) Schematic illustration of Se@LNT preparation. (B) TEM analysis: B1, representative TEM image of SeNPs (scale bar: 100 nm); B2-B3, representative TEM images of Se@LNT (scale bars: 100 nm and 20 nm). (C) Elemental mapping of Se@LNT showing the distribution of carbon (red), oxygen (yellow), and selenium (blue) (scale bar: 20 nm). (D) Particle size distribution of Se@LNT. (E) Zeta potential of SeNPs, LNT, and Se@LNT. (F) XPS spectra of LNT and Se@LNT. (G) Binding energy of O 1s in Se@LNT. (H) Binding energy of Se 3d in Se@LNT. (I-J) Stability of Se@LNT in PBS and DMEM containing FBS (10%). (K) ABTS radical scavenging activity of Se@LNT. (L) TEM images of Se@LNT: L1, after incubation in PBS (pH 7.4); L2, after incubation in PBS (pH 6.8) containing H_2_O_2_ (100 μM). Scale bars: 50 nm. (*n* = 3)

To overcome the limitations of selenium delivery, Se@LNT composite nanoparticles were successfully synthesized via a redox reaction between sodium selenite (Na_2_SeO_3_) and ascorbic acid in the presence or absence of LNT, yielding either lentinan-functionalized selenium nanoparticles (Se@LNT) or unmodified elemental SeNPs (Figure 2A). Transmission electron microscopy (TEM) analysis showed that unmodified SeNPs exhibited irregular morphologies and heterogeneous size distributions, whereas LNT-coated Se@LNT nanoparticles displayed well-dispersed and uniformly spherical structures (Figure 2B), suggesting that LNT modification significantly enhanced the structural uniformity and colloidal stability of SeNPs 40, 41. Moreover, previous studies have reported that LNT, as a natural bioactive polysaccharide, possesses excellent biocompatibility and intrinsic antioxidant properties. Its surface coating on SeNPs can further improve the antioxidant capacity and biosafety of the nanomaterial, providing a promising foundation for biomedical applications 42, 43.

Elemental mapping by energy-dispersive X-ray spectroscopy (EDS) further confirmed the homogeneous distribution of selenium within the Se@LNT structure, verifying successful lentinan-selenium composite formation (Figure 2C). Dynamic light scattering (DLS) analysis demonstrated a hydrodynamic diameter of approximately 208 nm for unmodified SeNPs, which notably decreased to 112 nm, accompanied by a narrower size distribution upon lentinan modification in Se@LNT nanoparticles (Figure 2D; Figures S8-S9). Additionally, the zeta potential of Se@LNT nanoparticles was -19.6 mV, indicating a stronger negative surface charge than either free LNT or unmodified SeNPs alone (Figure 2E; Figure S10). In addition, the chemical bonding states of Se@LNT were systematically characterized by X-ray photoelectron spectroscopy (XPS) (Figure 2F). The high-resolution O 1s spectrum of LNT displayed typical peaks at 531.08 eV (C=O/C-OH) and 532.52 eV (C-O) (Figure 2G; Figure S11). After selenium modification, the O 1s spectrum of Se@LNT retained these characteristic peaks and exhibited a new signal at 530.24 eV, which can be attributed to the formation of Se-O bonds, indicating covalent interactions between selenium and hydroxyl groups on the polysaccharide surface. Further deconvolution of the Se 3d spectrum in Se@LNT (Figure 2H) revealed two distinct doublet components. The peaks at 58.36 eV (Se 3d_5_/_2_) and 59.11 eV (Se 3d_3_/_2_) were assigned to Se-O/Se(IV)-OH structures, while the peaks at 54.80 eV and 55.60 eV corresponded to the 3d orbitals of elemental selenium (Se^0^). These results suggest that upon complexation with LNT, surface selenium species in Se@LNT undergo coordination with oxygen-containing groups to form Se-O bonds 43, 44. Notably, the formation of Se-O bonds not only increases the surface polarity of the nanoparticles but also enhances their colloidal stability via steric hindrance. Altogether, the XPS findings provide strong electronic-structural evidence supporting the successful construction of the Se@LNT nanocomposite. The biological activity of selenium is highly dependent on its chemical form 45. Inorganic selenium (e.g., Na_2_SeO_3_) must undergo reduction to selenides (Se^2-^) in the gastrointestinal tract before absorption, but its high oxidative potential results in dose-dependent toxicity and low bioavailability. Organic selenium (e.g., selenomethionine, SeMet; selenocysteine, SeCys_2_) exhibits lower toxicity but suffers from poor solubility, complex metabolic pathways, and nonspecific accumulation in non-target organs 20, 22, 27, 28, 45. In contrast, Se@LNT enables responsive ROS-triggered release of active selenium through Se-O coordination bonds, which is metabolically converted to SeCys_2_ for efficient incorporation into the catalytic center of GPX1. Its nanoscale dimension and LNT modification significantly enhance bioavailability while reducing toxicity thresholds. Owing to these advantages, the cytotoxicity profiles of various selenium formulations were assessed in OFs. As shown in Figure S12, (Na_2_SeO_3_ exhibited the highest cytotoxicity due to the reactive nature of free selenite, while SeMet and SeCys_2_ showed toxicity at relatively high concentrations (>10 μM). In contrast, Se@LNT demonstrated a markedly wider safety margin due to the stabilizing effect of LNT on selenium release, and was therefore selected for subsequent studies.

To systematically evaluate the stability and bioactivity of Se@LNT, we first monitored its particle size over 7 days under simulated physiological conditions, including PBS (pH 7.4) and DMEM supplemented with FBS (10%). As shown in Figures 2I and 2J, the particle size of Se@LNT remained consistently stable in both media, demonstrating excellent colloidal stability suitable for systemic administration. Further analysis using the ABTS radical scavenging assay revealed a dose-dependent antioxidant activity of Se@LNT at concentrations of 2.5 and 5.0 µM. The radical scavenging capacity was significantly higher in Se@LNT-treated groups compared to the untreated control (Figure 2K), indicating its robust potential to combat oxidative stress. In TED patients, the enclosed orbital cavity, inflammatory adipose infiltration, and hyaluronic acid accumulation contribute to the formation of a pathological acidic microenvironment. Environmental factors such as smoking and high altitude further reduce oxygen availability in periorbital tissues, amplifying ROS generation and glycolysis, thereby reinforcing a vicious cycle of hypoxia, acidosis, and oxidative stress 46. To evaluate the structural stability and environmental responsiveness of Se@LNT under pathological conditions, we established a dual-responsive degradation model by incubating Se@LNT in simulated systemic circulation (PBS, pH 7.4), mildly acidic microenvironment (PBS, pH 6.8), and oxidative stress conditions (PBS, pH 6.8 with H_2_O_2_, 100 μM). TEM-based dynamic monitoring (Figure 2L; Figure S13) revealed that Se@LNT maintained its structural integrity under physiological conditions (pH 7.4). In mildly acidic PBS (pH 6.8), moderate degradation of the LNT shell was observed at 48 hours, while the selenium core remained intact. In contrast, under oxidative conditions, Se@LNT exhibited rapid structural disruption within 6 hours and complete dissociation by 24 hours. This environment-dependent degradation pattern is likely attributable to the specific ROS-responsive nature of Se-O bonds. These results confirm the dual-stable degradation behavior of Se@LNT, highlighting its potential for stimulus-triggered release under oxidative stress while maintaining stability under normal physiological conditions. To directly verify the metabolic transformation of Se@LNT within OFs, HPLC-ICP-MS was employed to track selenium species over time. As shown in the overlaid spectra (Figure S14), Se@LNT-treated cells exhibited a time-dependent accumulation of SeCys_2_, accompanied by mild elevations in MeSeCys and Se(IV). In contrast, control cells lacked significant SeCys_2_ signals, confirming that the intracellular environment enables Se@LNT bioconversion into SeCys_2_. This supports the hypothesis that Se@LNT serves as a metabolic precursor for bioactive selenocompounds, enabling targeted selenoprotein synthesis under oxidative stress 29. Confocal laser scanning microscopy (CLSM) further confirmed efficient internalization of Se@LNT into OFs via endocytosis, establishing a foundation for targeted delivery. After a 2-hour incubation with Cy5-labeled selenium nanoparticles (Cy5-Se@LNT), significant intracellular fluorescence was detected (Figure S15), validating cellular uptake. Selenium is known to upregulate selenoproteins, particularly GPX1 and SELENOP 20. Considering the critical roles of selenium in the function of various selenoproteins, qPCR analysis was conducted to evaluate gene expression changes in OFs following Se@LNT treatment (Figure S16). Results showed a dose-dependent upregulation of GPX1, GPX4, and SELENOP expression. In summary, our results suggest that Se@LNT modulates GPX1-mediated mitochondrial quality control, potentially interrupting the pathological interplay among hypoxia, acidosis, and ROS accumulation. Subsequent functional validation experiments have further supported the ability of Se@LNT to reprogram metabolic flux, providing mechanistic evidence for its protective role against oxidative stress and metabolic dysregulation in TED. These findings establish Se@LNT as a dual-modal delivery system, capable of both maintaining homeostatic protection and enabling stress-activated release, offering a promising strategy for the development of novel microenvironment-responsive targeted antioxidant therapies.

### Se@LNT attenuates oxidative stress in TED

**Figure 3:**
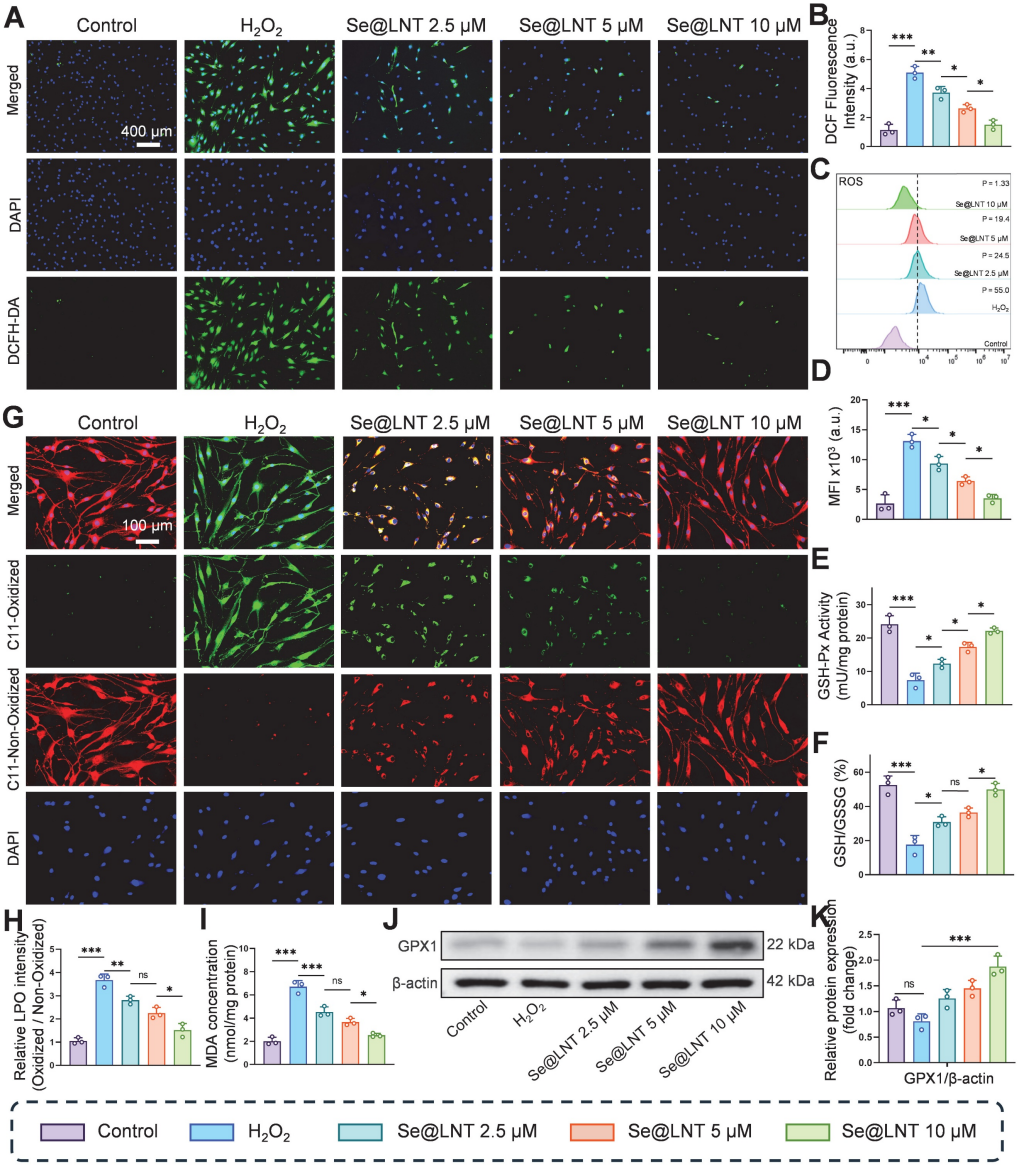
Evaluation of oxidative stress attenuation by selenium nanoparticles in TED. (A-B) DCFH-DA fluorescence staining and quantitative analysis of ROS levels (scale bar: 400 μm). (C-D) Flow cytometry analysis of ROS and corresponding quantification. MFI, mean fluorescence intensity. P, percentage of cells exhibiting a rightward fluorescence shift relative to the control group. (E) Measurement of GSH-Px activity. (F) GSH/GSSG ratio. (G-H) C11-BODIPY staining and quantification of lipid peroxidation (scale bar: 100 μm). (I) Quantification of MDA levels. (J-K) Western blot analysis of GPX1 expression and its quantification. (ns, no significance; *p < 0.05; **p < 0.01; ***p < 0.001; mean ± SE, *n* = 3).

The progression of inflammatory adipogenesis in TED is initiated by oxidative stress, primarily involving mitochondrial dysfunction and ROS accumulation 38. Clinically, elevated levels of ROS, along with abnormal accumulation of lipid peroxidation marker MDA and DNA oxidative damage marker 8-OHDG, have been confirmed in both serum and OFs of TED patients, indicating systemic oxidative injury 47. Se@LNT exerts antioxidant effects by upregulating GPX1, which scavenges ROS and lipid hydroperoxides, thereby maintaining redox homeostasis. DCFH-DA fluorescence staining demonstrated that H_2_O_2_ stimulation significantly increased ROS levels in OFs, while Se@LNT treatment suppressed ROS accumulation in a dose-dependent manner (Figures 3A-B). This trend was further validated by flow cytometry, where fluorescence intensity in the H_2_O_2_-treated group was markedly elevated compared to the control group, but was restored to basal levels upon treatment with Se@LNT (10 μM) (Figures 3C-D). These findings indicate that Se@LNT effectively mitigates oxidative stress by eliminating excessive ROS. H_2_O_2_ exposure reduced GSH-Px activity by 69.25% relative to the control group, while intervention with Se@LNT (10 μM) restored enzyme activity to 94.23%. Additionally, the GSH/GSSG ratio concurrently increased to 49.98% (Figures 3E-F), confirming the reconstruction of the antioxidant defense network via glutathione system repair. C11-BODIPY staining revealed a 3.48-fold increase in lipid peroxidation (LPO) intensity following H_2_O_2_ induction, which was significantly alleviated by Se@LNT (10 μM) treatment (Figures 3G-H). Similarly, MDA levels in the H_2_O_2_ group were 3.39 times higher than those in the control group, and were reduced by 63.15% following Se@LNT intervention (Figure 3I), indicating that Se@LNT interrupts lipid peroxidation chain reactions by scavenging lipid radicals. Western blot analysis showed that Se@LNT pretreatment increased GPX1 protein expression compared to the H_2_O_2_ group, with a concentration-dependent trend (Figures 3J-K). These results suggest that Se@LNT enhances cellular antioxidant capacity by promoting GPX1 protein abundance through metabolic conversion to SeCys_2_.

**Figure 4:**
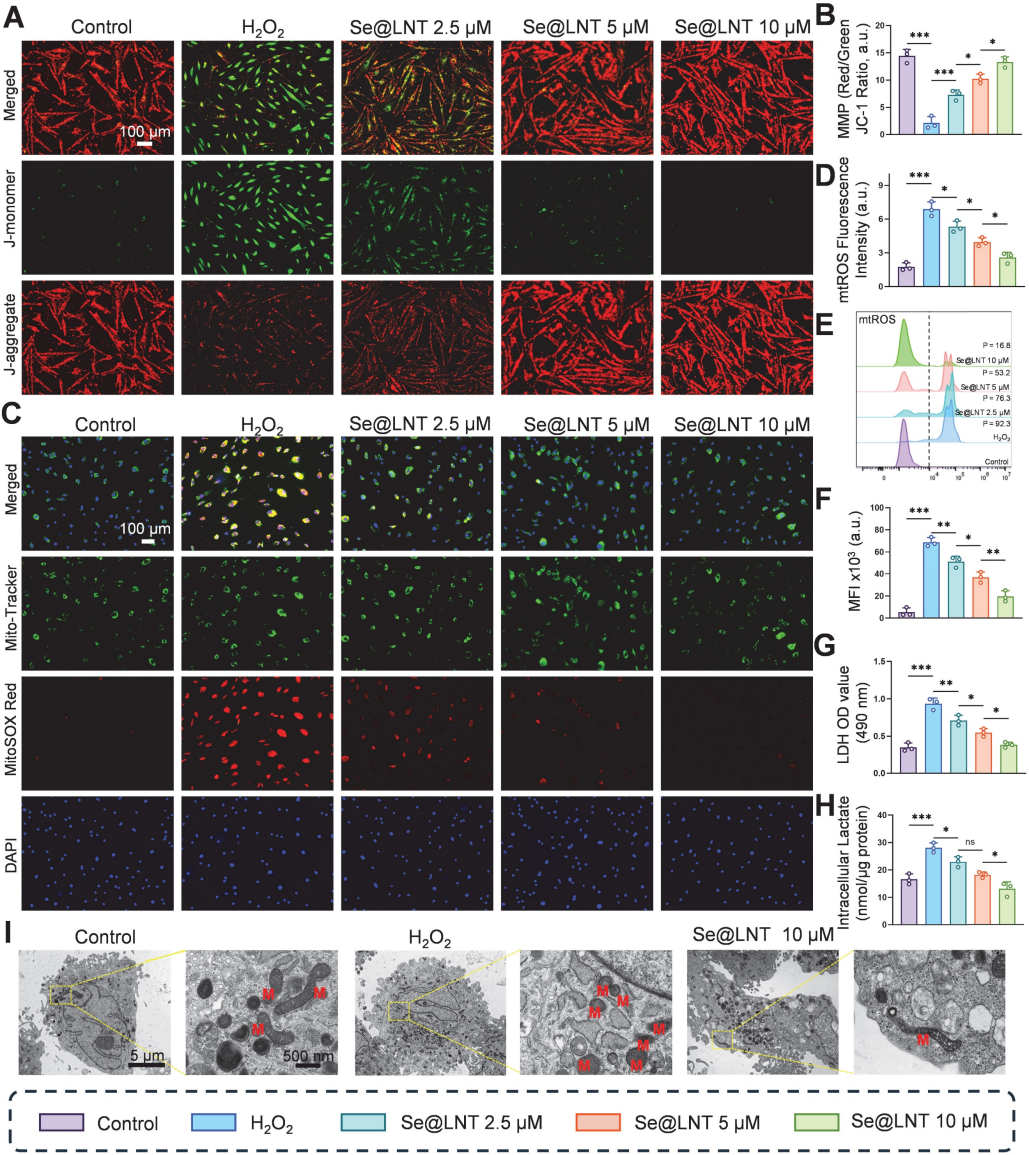
Evaluation of Se@LNT effects on mitochondrial function and energy metabolism. (A-B) Mitochondrial membrane potential staining and quantification (scale bar: 100 μm). (C-D) Localization and quantification of mtROS (scale bar: 100 μm). (E-F) Flow cytometric analysis and quantification of mtROS. MFI, mean fluorescence intensity. P, percentage of cells exhibiting a rightward fluorescence shift relative to the control group. (G) LDH activity assay. (H) Intracellular lactate concentration. (I) TEM images showing ultrastructural morphology; mitochondria labeled as M. Left panel scale bar: 5 μm; right panel scale bar: 500 nm. (ns, no significance; *p < 0.05; **p < 0.01; ***p < 0.001; mean ± SE, *n* = 3)

This study elucidates the protective effects of Se@LNT on OFs by analyzing mitochondrial function and metabolic reprogramming. JC-1 staining revealed that MMP was decreased following H_2_O_2_ exposure compared to the control group, whereas treatment with Se@LNT (10 μM) significantly restored MMP levels, indicating its potential role in stabilizing mitochondrial function and interrupting oxidative damage cascades (Figures 4A-B). In addition to general ROS detection by DCFH-DA, we applied MitoSOX Red to specifically monitor mitochondrial superoxide (mtROS) levels, minimizing pH-dependent artifacts 48. MitoSOX Red staining showed that mtROS levels in the H_2_O_2_ group were 3.93 times higher than those in the control group, while Se@LNT (10 μM) treatment effectively reduced mtROS to baseline levels (Figures 4C-D; Figure S17). This finding was further validated by flow cytometry, which showed a 12.9-fold increase in mtROS fluorescence intensity in the H_2_O_2_ group, reduced by 71.4% following Se@LNT intervention (Figures 4E-F), confirming the nanoparticle's potent mtROS scavenging capability. These orthogonal results reinforce the specificity of Se@LNT in restoring mitochondrial redox balance. H_2_O_2_ treatment led to a 2.67-fold increase in lactate dehydrogenase (LDH) release compared to the control group, while Se@LNT (10 μM) reduced LDH release to baseline (Figure 4G).

Intracellular lactate levels also increased to 1.7 times that of the control group, but were significantly reduced by 53.5% following Se@LNT treatment (Figure 4H), indicating its efficacy in mitigating excessive glycolysis and correcting metabolic reprogramming. To further evaluate cellular energy metabolism, Seahorse XF96 extracellular flux analysis was performed and presented in Figure S18. H_2_O_2_ exposure significantly impaired mitochondrial respiration, evidenced by reductions in basal respiration, ATP production, and spare respiratory capacity. Se@LNT partially restored these parameters, suggesting a protective effect on mitochondrial function (Figure S18A-F). In parallel, extracellular acidification rate (ECAR) analysis revealed that H_2_O_2_ markedly elevated glycolysis and glycolytic capacity, while Se@LNT treatment suppressed this increase and restored glycolytic parameters toward baseline levels Figure S18G-K). These findings indicate that Se@LNT not only preserves mitochondrial oxidative phosphorylation but also alleviates pathological glycolytic reprogramming under oxidative stress conditions. TEM analysis revealed that, under standard *in vitro* culture conditions, OFs isolated from TED patients exhibited intact mitochondrial structures characterized by clearly defined cristae. This observation likely reflects a temporary normalization due to the absence of continuous oxidative and inflammatory stressors present *in vivo*. However, upon H_2_O_2_ stimulation, significant morphological changes were observed, characterized by mitochondrial condensation, rounding, cristae disruption, and swelling, confirming the intrinsic susceptibility of TED-derived OFs to oxidative stress. Notably, Se@LNT treatment (10 μM) substantially improved mitochondrial morphology and partially restored cristae integrity (Figure 4I). These findings further support the rationale of targeting oxidative stress-induced mitochondrial damage as a clinically relevant therapeutic approach, consistent with established disease models of TED pathogenesis 7, 38, 39. Collectively, Se@LNT exerts therapeutic efficacy by mitigating mitochondrial oxidative injury and restoring metabolic homeostasis, underscored by the functional recovery of mitochondrial respiration and normalization of glycolytic flux.

### Se@LNT attenuates pathological adipogenic differentiation of OFs

**Figure 5:**
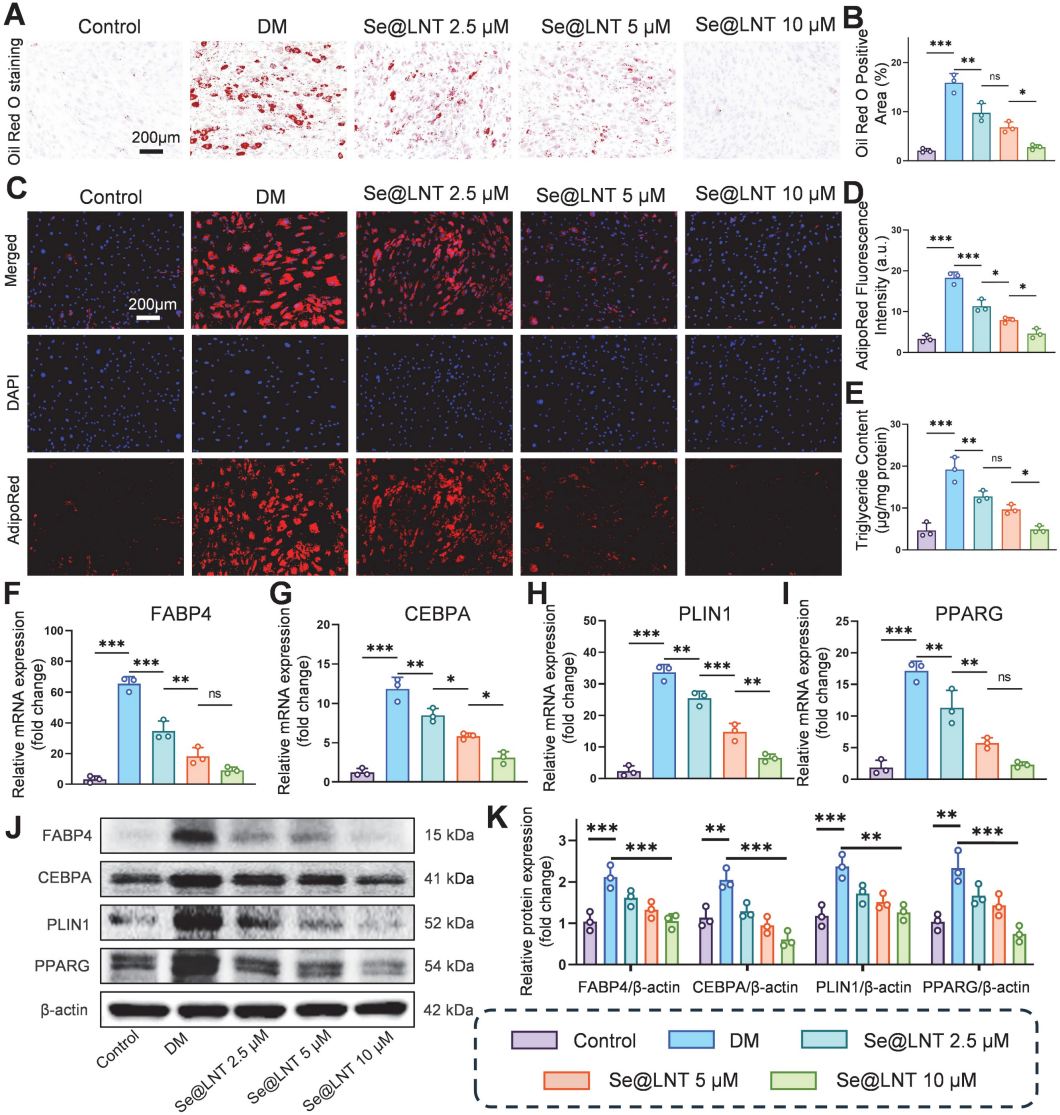
Investigation of selenium nanoparticles in suppressing adipogenic differentiation of OFs. (A-B) Oil Red O staining and quantification of adipogenic differentiation (scale bar: 200 μm). (C-D) AdipoRed fluorescence staining and quantification (scale bar: 200 μm). (E) Intracellular triglyceride content measurement. (F-I) mRNA expression analysis of key adipogenic genes. (J-K) Western blot analysis and quantification of adipogenic marker proteins. (ns, no significance; *p < 0.05; **p < 0.01; ***p < 0.001; mean ± SE, *n* = 3)

Oxidative stress promotes aberrant differentiation of OFs into adipocytes, exacerbating orbital fat expansion in TED 49. Mechanistically, thyroid hormone imbalance and TRAb stimulate the PERK/ATF4/CHOP axis, leading to overexpression of PPARG and CEBPA, which promotes lipid droplet formation and adipocyte differentiation 11. Concurrently, ER stress upregulates the chaperone protein BiP, which enhances FABP4-mediated fatty acid uptake; BiP silencing has been shown to reduce lipid droplet area 12. After 10 days of adipogenic induction using differentiation medium (DM), Oil Red O staining revealed marked lipid droplet accumulation, which was reduced by 82.7% following Se@LNT (10 μM) treatment (Figures 5A-B). AdipoRed fluorescence staining corroborated this trend, with fluorescence intensity in the DM group 5.5 times higher than that in the control group; Se@LNT treatment reduced this signal to baseline levels (Figures 5C-D). ELISA quantification demonstrated a 4.09-fold increase in triglyceride content in the DM group compared to the control group, while Se@LNT treatment reduced this by 74.7% (Figure 5E), indicating a dose-dependent suppression of lipid accumulation and inhibition of adipogenic differentiation. QPCR analysis showed that DM treatment upregulated the mRNA expression of key adipogenic transcription factors FABP4, CEBPA, PLIN1, and PPARG by 19.2-fold, 8.4-fold, 13.4-fold, and 8.3-fold, respectively, compared to the control group (Figures 5F-I). Se@LNT (10 μM) intervention led to reductions of 86.0%, 73.7%, 80.7%, and 86.7% in the expression of FABP4, CEBPA, PLIN1, and PPARG, respectively. Western blot analysis confirmed these findings at the protein level, showing significant upregulation in the DM group and marked downregulation following Se@LNT treatment (Figures 5J-K), demonstrating that Se@LNT suppresses adipogenic differentiation by inhibiting the regulatory network at both transcriptional and translational levels. These results highlight ER stress-induced lipid metabolic dysregulation as a central node in TED-related adipogenesis, and support the role of Se@LNT in reversing this pathological process.

**Figure 6:**
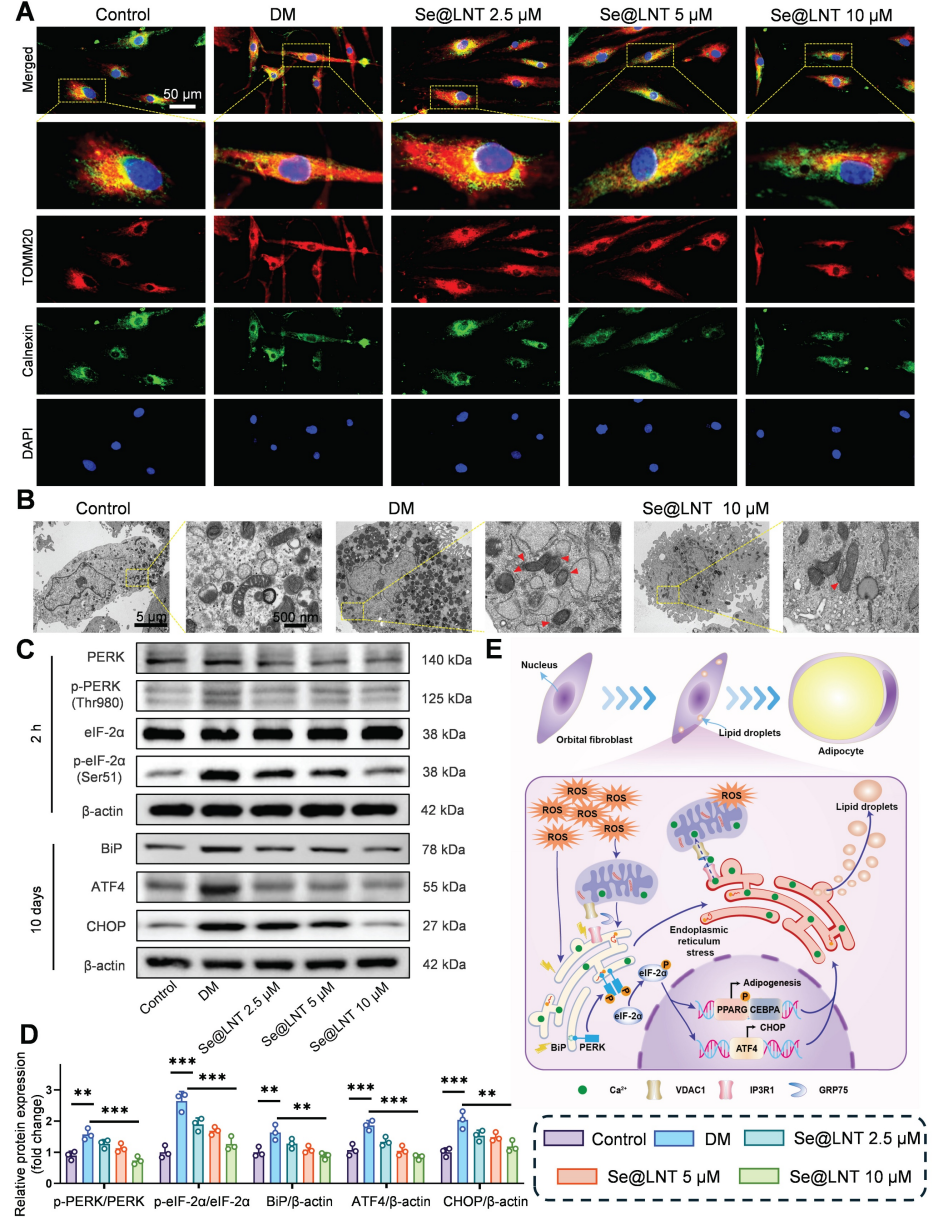
Mechanistic study of Se@LNT in regulating ERS to inhibit adipogenic differentiation. (A) Immunofluorescence colocalization analysis of Calnexin and TOMM20 (scale bar: 50 μm). (B) TEM analysis showing MAM structures indicated by red triangles. Left panel scale bar: 5 μm; right panel scale bar: 500 nm. (C-D) Western blot analysis and quantification of ER stress-related proteins. (E) Schematic model illustrating the molecular mechanism by which Se@LNT regulates ER stress. (**p < 0.01; ***p < 0.001; mean ± SE, *n* = 3)

Disruption of calcium signaling and metabolic substrate exchange between the ER and mitochondria ultimately drives metabolic reprogramming of OFs toward an adipogenic phenotype. To elucidate the role of MAMs in inflammatory adipogenesis, we first investigated their mechanistic involvement. After 10 days of adipogenic induction, colocalization analysis of Calnexin (ER marker) and TOMM20 (mitochondrial marker) revealed a marked increase in MAM formation in the DM group, indicating that ER stress aggravates mitochondrial damage through aberrant ER-mitochondria coupling (Figure 6A). Treatment with Se@LNT (10 μM) reduced the colocalization coefficient by 60.8% (Figure S19), suggesting that Se@LNT alleviates ER-mitochondria interaction disorder by restoring MAM homeostasis. TEM further showed that cells in the DM group exhibited ER dilation, accumulation of autophagosomes and lipid droplets, and disrupted mitochondrial cristae, while Se@LNT (10 μM) treatment restored ER morphology, reduced lipid droplet content, and improved mitochondrial cristae density (Figure 6B). Western blot analysis confirmed that ER stress markers including phosphorylated PERK (Thr980), phosphorylated eIF2α (Ser51), BiP, ATF4, and CHOP were significantly upregulated in the DM group compared to the control group, whereas Se@LNT (10 μM) pretreatment markedly suppressed their expression (Figures 6C-D). These results indicate that Se@LNT inhibits ER stress by suppressing the PERK-eIF2α signaling pathway and blocking the UPR. The proposed mechanism is illustrated in the schematic model (Figure 6E): During adipogenic differentiation of TED-derived OFs, ER stress leads to abnormal MAM coupling, resulting in mitochondrial calcium overload and ROS burst, which activates the PERK pathway and reprograms transcriptional activity toward adipogenesis. Collectively, Se@LNT suppresses pathological adipogenic differentiation by targeting GPX1 to scavenge ROS, restore calcium homeostasis at MAMs, and inhibit the ER stress cascade.

### Se@LNT inhibit MAMs formation and alleviate ERS

**Figure 7:**
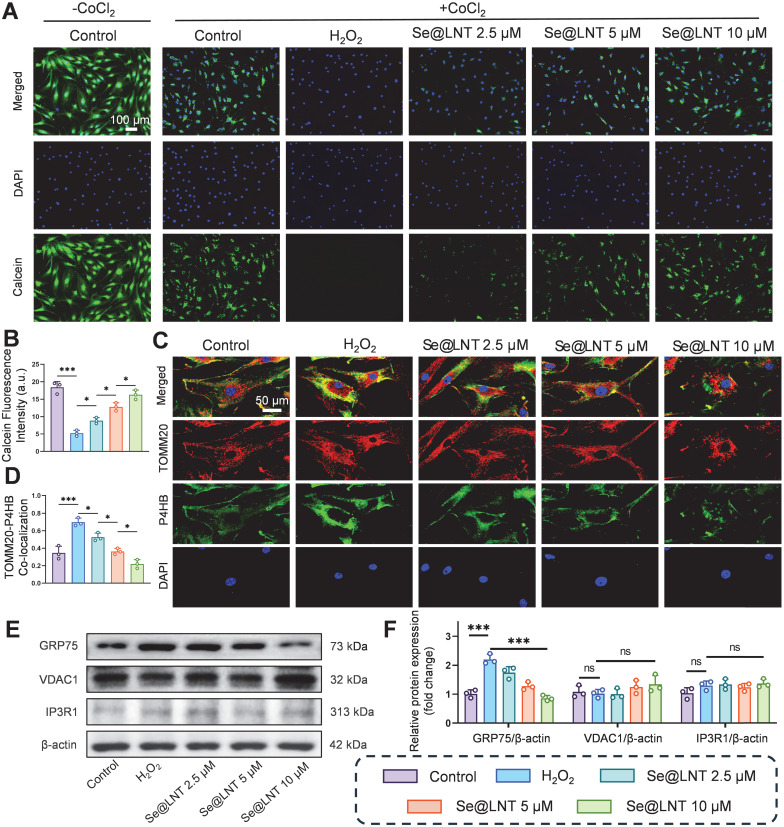
Mechanistic investigation of Se@LNT in regulating MAMs. (A-B) Assessment and quantification of MPTP opening (scale bar: 100 μm). (C-D) Immunofluorescence colocalization of mitochondria and ER, with quantitative colocalization analysis (scale bar: 50 μm). (E-F) Western blot analysis and quantification of MAMs-associated proteins. (ns, no significance; *p < 0.05; ***p < 0.001; mean ± SE, n = 3)

As the core interface for inter-organelle communication, MAMs regulate calcium homeostasis and metabolic substrate exchange through the IP3R1-GRP75-VDAC1 complex 14. GRP75, a member of the HSP70 family, serves as a molecular tether protein that directly maintains calcium signaling balance 50, 51. In age-related macular degeneration, aberrant MAM coupling accelerates lipid droplet accumulation and oxidative stress, contributing to pathological remodeling of the macula 13. Although the role of MAMs in metabolic diseases has been partially elucidated, their regulatory mechanisms in TED remain unclear. To investigate the modulatory effect of Se@LNT on MAMs in TED, we assessed MPTP opening, a hallmark of mitochondrial calcium overload resulting from abnormal MAM anchoring. In this assay, we used CoCl_2_ as a calcium-mimicking agent to chemically induce MPTP opening under stress conditions, as it is known to disrupt mitochondrial calcium homeostasis and mimic hypoxic stress 52, 53. A CoCl_2_-free baseline control group was included to represent normal mitochondrial integrity, which exhibited the highest Calcein fluorescence intensity. All other groups, including the H_2_O_2_ and Se@LNT-treated conditions, were co-treated with CoCl_2_ to standardize the induction of MPTP opening across conditions. Following H_2_O_2_ stimulation, MPTP opening significantly increased, whereas treatment with Se@LNT (10 μM) restored fluorescence intensity to levels comparable to the control group, indicating protection of mitochondrial membrane integrity through inhibition of aberrant MPTP opening (Figures 7A-B). Colocalization analysis of TOMM20 and P4HB (ER marker) showed that MAM formation increased by 1.01-fold in the H_2_O_2_ group compared to the control group, while Se@LNT (10 μM) significantly reduced the colocalization coefficient (Figures 7C-D), suggesting that Se@LNT mitigates ER-mitochondria interaction disorder by modulating MAM coupling. Western blot analysis revealed a marked upregulation of GRP75 expression following H_2_O_2_ induction, which was significantly downregulated by Se@LNT treatment (10 μM) (Figures 7E-F). In contrast, expression levels of VDAC1 and IP3R1 remained unchanged, indicating that Se@LNT specifically targets GRP75 to inhibit excessive MAM formation. Correlation analysis using the GEO dataset (GSE58331) further confirmed a significant negative correlation between GPX1 and GRP75 (r = -0.36, *p* = 0.01), a weak positive correlation with VDAC1 (r = 0.20, *p* = 0.05), and no correlation with IP3R1 (Figure S20). To further validate whether GRP75 downregulation by Se@LNT is dependent on GPX1 activity, we performed siRNA-mediated knockdown of GPX1 in H_2_O_2_-treated orbital fibroblasts. As shown in Figure S21, Se@LNT failed to suppress GRP75 expression in the presence of GPX1 silencing, whereas the effect was preserved in cells transfected with negative control siRNA. These findings are consistent with previous studies showing that redox-regulating selenoproteins such as GPX4 modulate MAM integrity and ER stress via GRP75 and PERK signaling 54-56, supporting the role of GPX1 as an upstream redox modulator of MAM homeostasis. These findings suggest that GPX1 regulates MAM homeostasis primarily through downregulation of GRP75, thereby disrupting the ER stress cascade. Although biochemical isolation of MAM fractions was not feasible due to limited primary OFs, future studies using fractionation-based proteomics will be valuable to further validate these findings. In summary, Se@LNT inhibits GRP75-mediated aberrant MAM coupling, preserves ER-mitochondrial calcium signaling balance, and alleviates pathological adipogenic differentiation in OFs. Targeting the MAM interface to restore calcium and redox homeostasis represents a critical therapeutic breakthrough in halting the progression of TED.

### Se@LNT inhibit the cGAS-STING pathway and downstream inflammatory responses

**Figure 8:**
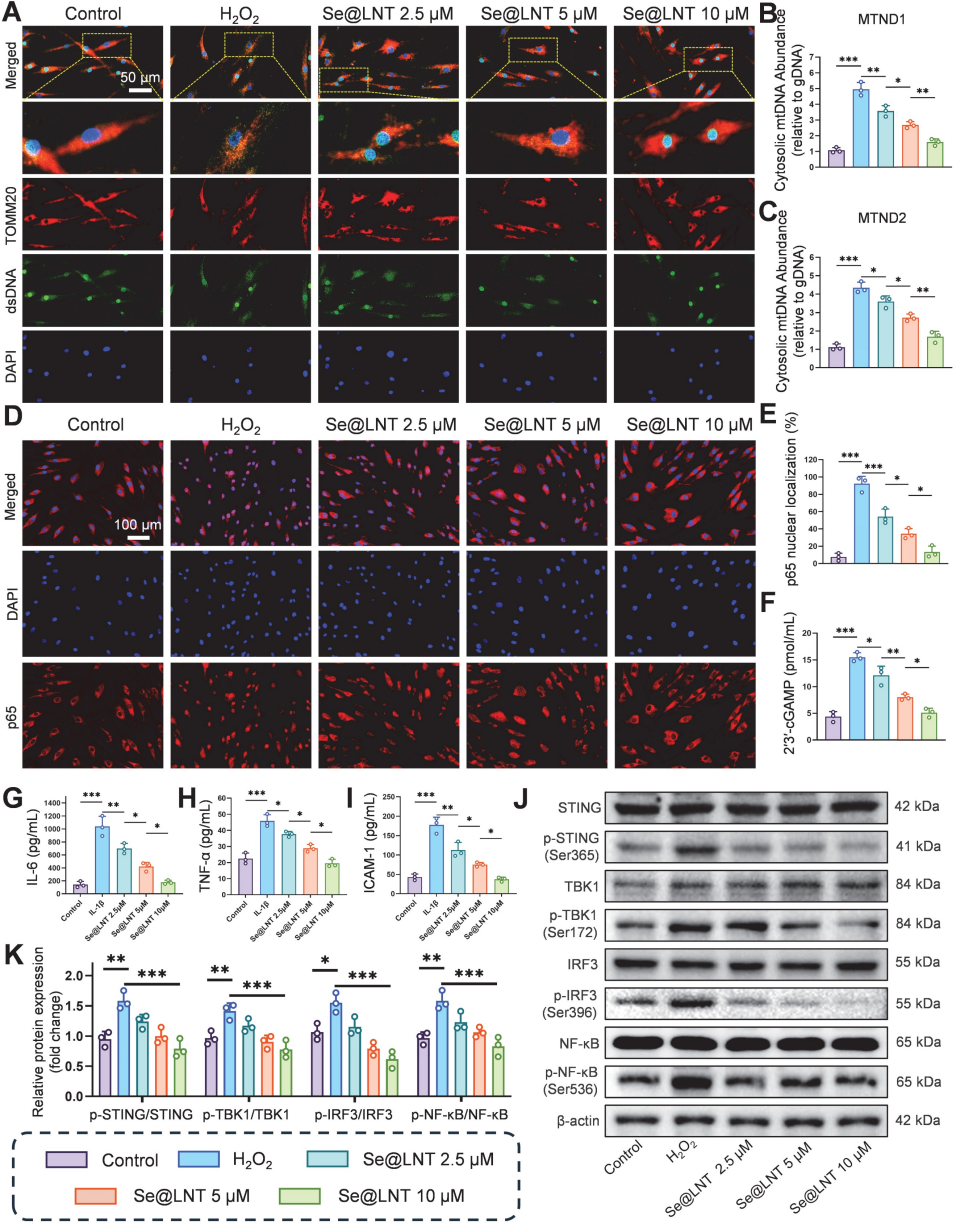
Mechanistic investigation of Se@LNT in suppressing cGAS-STING signaling and inflammatory responses. (A) Immunofluorescence analysis of dsDNA and TOMM20 colocalization (scale bar: 50 μm). (B-C) qPCR quantification of cytosolic mtDNA (MTND1 and MTND2). (D-E) Immunofluorescence and quantification of nuclear translocation of p65 (scale bar: 100 μm). (F) Measurement of 2′3′-cGAMP levels. (G-I) ELISA detection of inflammatory cytokines (IL-6, TNF-α, and ICAM-1). (J-K) Western blot analysis and quantification of IRF3/NF-κB pathway proteins. (ns, no significance; *p < 0.05; **p < 0.01; ***p < 0.001; mean ± SE, *n* = 3)

Calcium overload triggers the opening of MPTPs, leading to the release of mtDNA and subsequent activation of the cGAS-STING pathway, which drives inflammatory cascades and innate immune responses 57, 58. Leaked mtDNA is recognized by cytosolic cGAS, initiating STING-dependent IRF3/NF-κB signaling that promotes the secretion of inflammatory cytokines and polarization of Th1/Th17 cells 59, 60. This MAMs-mediated vicious cycle of oxidative stress and inflammation further exacerbates adipose tissue expansion. Following H_2_O_2_ stimulation, the colocalization of dsDNA with TOMM20 was markedly reduced (Figure 8A), indicating mitochondrial membrane disruption and mtDNA leakage into the cytosol. Pretreatment with Se@LNT restored dsDNA-TOMM20 colocalization in a concentration-dependent manner, suggesting that Se@LNT preserves mitochondrial integrity and prevents mtDNA release. Fluorescence staining for 8-OHDG revealed a 3.53-fold increase in mitochondrial DNA oxidative damage in the H_2_O_2_ group compared to the control group, which was reduced to baseline levels after Se@LNT treatment (10 μM) (Figure S22). QPCR analysis showed that cytosolic levels of mtDNA marker genes MTND1 and MTND2 increased by 4.62-fold and 3.88-fold, respectively, in the H_2_O_2_ group versus the control group, while Se@LNT treatment (10 μM) restored both to baseline levels (Figures 8B-C), confirming that Se@LNT inhibits cGAS-STING pathway activation by preventing mtDNA leakage. Analysis of p65 nuclear localization demonstrated a dramatic increase from 7.63% to 92.17% upon H_2_O_2_ exposure, which was reduced to 13.37% following Se@LNT treatment (Figures 8D-E). The cGAS-STING pathway product 2′3′-cGAMP was elevated 3.55-fold in the H_2_O_2_ group relative to the control group, and was normalized after Se@LNT intervention (Figure 8F). ELISA results in an IL-1β-induced inflammatory model showed that levels of IL-6, TNF-α, and ICAM-1 increased by 7.32-fold, 2.04-fold, and 4.12-fold, respectively, compared to the control group, and were significantly reduced to baseline following Se@LNT treatment (10 μM) (Figures 8G-I).

Western blot analysis revealed that H_2_O_2_ markedly increased the expression of p-STING/STING, p-TBK1/TBK1, p-IRF3/IRF3, and p-NF-κB/NF-κB, all of which were downregulated by Se@LNT (10 μM) (Figures 8J-K). Together, these findings demonstrate that Se@LNT suppresses activation of the cGAS-STING pathway by preventing mtDNA release, thereby inhibiting the downstream TBK1-IRF3 and NF-κB inflammatory cascades, ultimately alleviating inflammatory adipogenic differentiation in TED-derived OFs.

### Transcriptomic analysis of Se@LNT intervention

**Figure 9:**
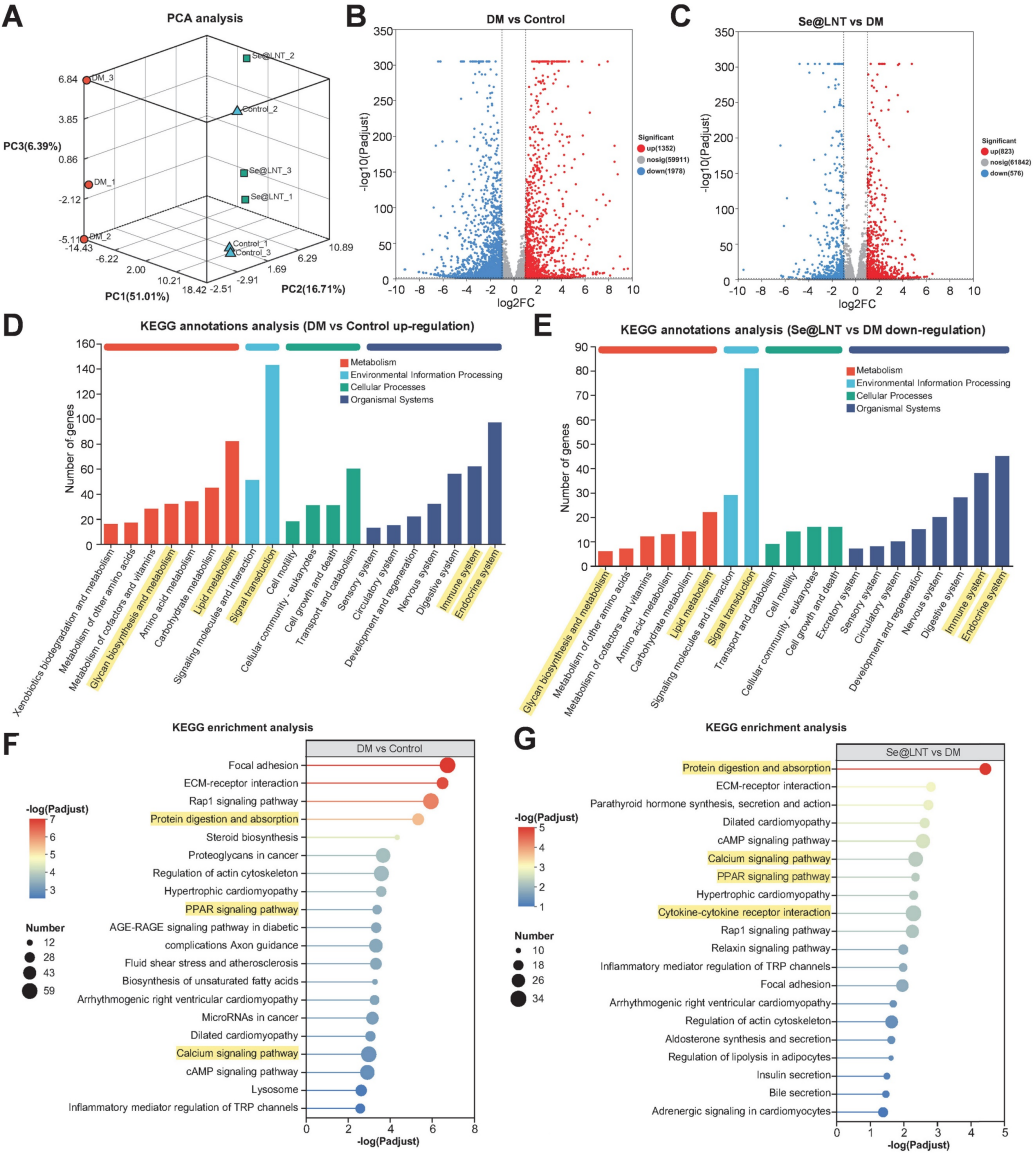
Transcriptional reprogramming by selenium nanoparticles reverses adipogenic differentiation in TED. (A) Three-dimensional PCA illustrating global transcriptomic differences among the control, DM (adipogenic differentiation), and Se@LNT intervention groups. (B) Volcano plot of DEGs between DM and control groups, identifying 1,352 upregulated and 1,978 downregulated genes (threshold: |log2FC| ≥ 1 and adjusted *p* < 0.05. (C) Se@LNT-mediated reversal of DM-induced DEGs, with 823 upregulated and 576 downregulated genes (threshold as in B). (D) KEGG functional annotation of genes upregulated in DM vs. control. (E) KEGG annotation of genes downregulated in Se@LNT vs. DM groups. (F) KEGG pathway enrichment analysis of upregulated genes in DM vs. control. (G) KEGG pathway enrichment analysis of downregulated genes in Se@LNT vs. DM.

Transcriptome sequencing revealed that Se@LNT reverses adipogenic differentiation in TED-OFs through coordinated regulation of multiple signaling pathways. Three-dimensional principal component analysis (PCA) demonstrated clear spatial separation among control, DM, and Se@LNT-treated groups at the transcriptomic level (Figure 9A), indicating that both DM induction and Se@LNT treatment exerted global regulatory effects on gene expression profiles in TED-OFs. Volcano plot analysis identified 1,352 upregulated and 1,978 downregulated genes in the DM group compared to control (Figure 9B), whereas Se@LNT treatment reversed these changes, upregulating 823 and downregulating 576 genes relative to DM (Figure 9C). These results suggest that Se@LNT reshapes transcriptomic homeostasis through multi-target interaction, overcoming the pathway limitations of conventional antioxidants. KEGG pathway enrichment analysis revealed that genes upregulated in the DM group were significantly enriched in lipid metabolism, signal transduction, and immune system pathways (Figure 9D), while Se@LNT treatment notably suppressed the expression of genes involved in these pathways (Figure 9E). Further enrichment analysis showed that the DM group exhibited activation of pathways such as protein digestion and absorption, PPAR signaling pathway, and calcium signaling pathway (Figure 9F), consistent with earlier findings of GPX1-mediated restoration of MAMs calcium homeostasis. Importantly, Se@LNT not only reduced the activity of these pathways but also inhibited cytokine-cytokine receptor interaction signaling (Figure 9G), underscoring its dual roles in metabolic reprogramming and inflammation suppression. A Venn diagram identified 975 overlapping differentially expressed genes between the DM vs. control and Se@LNT vs. DM comparisons (Figure S23A), with expression patterns shown in the corresponding heatmap (Figure S23B). Clustering trend analysis indicated that subcluster_1 (559 genes), including GPX1, was significantly downregulated in the DM group but partially restored by Se@LNT; conversely, subcluster_2 (365 genes) was upregulated in the DM group and suppressed upon Se@LNT treatment (Figures S23C-D). These transcriptomic findings confirm that Se@LNT blocks pathological adipogenic differentiation in TED-OFs by orchestrating metabolic reprogramming and inflammatory inhibition at the molecular level. To validate the transcriptomic findings at the molecular level, we focused on the “Protein digestion and absorption” pathway, which was significantly enriched in both the DM vs. control and Se@LNT vs. DM comparisons. By intersecting the differentially expressed genes in these two comparisons with genes in subcluster_2 (those upregulated in DM and suppressed by Se@LNT), seven overlapping genes were identified (Figure S24A). Among these, four genes (CPB1, COL4A1, ACE2, and ATP1B2) were selected for qPCR validation based on their functional relevance to ER stress and adipogenic remodeling. Consistent with the transcriptomic profiles, all four genes were significantly upregulated in the DM group and downregulated following Se@LNT treatment (Figures S24B-E). These results reinforce the reliability of our RNA-seq data and support the role of Se@LNT in modulating ER-associated transcriptional responses during adipogenic differentiation.

### Selenium nanoparticles restore immune homeostasis in a TED mouse model

**Figure 10:**
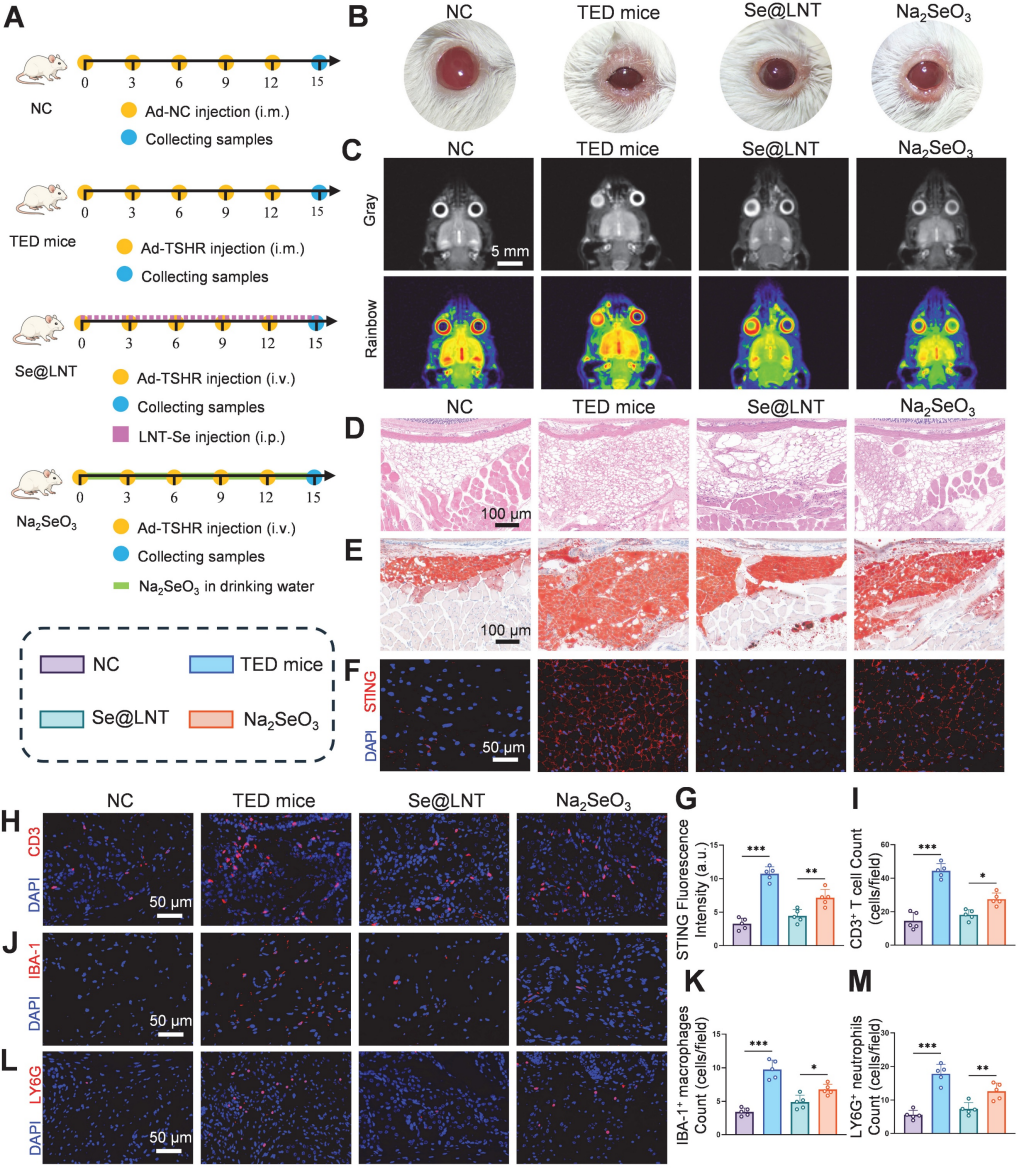
Se@LNT attenuates orbital adipose hyperplasia and immune dysregulation in TED mice via suppression of the STING inflammatory pathway. (A) Schematic of TED mouse model establishment and therapeutic regimen. (B) Comparative visualization of orbital morphology in mice. (C) Coronal MRI imaging with Gray/Rainbow pseudo-coloring (scale bar: 5 mm). (D) HE staining of orbital adipose tissue (scale bar: 100 μm). (E) Oil Red O staining of orbital adipose lipid deposition (scale bar: 100 μm). (F-G) STING immunofluorescence staining and quantification in orbital adipose tissue (scale bar: 50 μm). (H-M) Immunofluorescence staining and quantification of infiltrating immune cells: (H-I) CD3^+^ T cells, (J-K) IBA-1^+^ macrophages, and (L-M) LY6G^+^ neutrophils in periocular adipose tissue (scale bars: 50 μm). (*p < 0.05; **p < 0.01; ***p < 0.001; mean ± SE, *n* = 5)

Selenoprotein deficiency exacerbates immune dysregulation in TED. In selenium-depleted conditions, macrophages accumulate ROS, undergo extracellular matrix remodeling, and exhibit impaired migration with a tendency toward M1 polarization. Helper T cells (Th) also display an overactivated phenotype due to imbalanced Th1/Th17 differentiation triggered by selenium deficiency. Previous studies have demonstrated that selenium supplementation restores redox balance via GPX-mediated mechanisms, promoting regulatory T cell (Treg) expansion while inhibiting Th1/Th17 differentiation and reducing thyroid autoantibody levels 61, 62. To evaluate the immunomodulatory effects of selenium nanoparticles, four groups were established: NC, TED mice, Se@LNT, and Na_2_SeO_3_ (Figure 10A). In the TED mouse model, serum thyroid-stimulating hormone (TSH) levels were significantly reduced, while TRAb and thyroxine (T4) levels were elevated compared to controls (Figure S25). Hematoxylin and eosin (HE) staining of thyroid tissue confirmed follicular epithelial hyperplasia and lymphocytic infiltration in the TED group (Figure S26), validating successful disease modeling. Body weight monitoring revealed no significant differences among the four groups (Figure S27), indicating that the administered doses of Se@LNT and Na_2_SeO_3_ were non-toxic systemically. Se@LNT treatment significantly improved the orbital phenotype in TED mice. Compared to the TED group, which exhibited proptosis, eyelid edema, and widened palpebral fissures, mice in the Se@LNT group displayed nearly normal ocular appearance (Figure 10B). Magnetic resonance imaging (MRI) analysis further showed extraocular muscle hypertrophy and retrobulbar fibroadipose proliferation in TED mice, both of which were markedly ameliorated following Se@LNT intervention (Figure 10C; Figure S28).

Histological examination using HE and Oil Red O staining confirmed orbital adipose tissue expansion in the TED group, while lipid droplet accumulation was significantly reduced in the Se@LNT group compared to the Na_2_SeO_3_ group (Figures 10D-E; Figure S29). To further assess the ultrastructural changes in orbital fibroblasts *in vivo*, TEM was performed on orbital adipose tissues. In the NC group, mitochondria appeared structurally intact with well-defined cristae. In contrast, the TED group showed pronounced mitochondrial shrinkage, cristae loss, and the emergence of MAMs. Se@LNT treatment restored mitochondrial morphology and diminished MAM formation, while Na_2_SeO_3_ partially improved endoplasmic reticulum swelling but failed to fully resolve mitochondrial damage or MAM accumulation (Figure S30).

Immunofluorescence analysis revealed significant STING activation in the orbital adipose tissue of TED mice, whereas STING expression in the Se@LNT group was reduced by 37.8% compared to the Na_2_SeO_3_ group (Figures 10F-G). This aligns with previous findings on mtDNA leakage and cGAS pathway inhibition, and demonstrates the superior anti-inflammatory efficacy of selenium nanoparticles over traditional selenium agents by blocking innate immune activation. In parallel, ELISA analysis of serum inflammatory markers further confirmed this trend. Levels of IL-6, TNF-α, and ICAM-1 were significantly elevated in TED mice compared to controls, reflecting systemic inflammation. Se@LNT treatment markedly reduced all three markers compared to the Na_2_SeO_3_ group, indicating its stronger systemic anti-inflammatory effect (Figure S31). Infiltration of CD3^+^ T cells, accumulation of IBA-1^+^ macrophages, and LY6G^+^ neutrophils in eyelid tissues were markedly increased in the TED group. Se@LNT treatment significantly reduced these immune cell populations by 34.0%, 27.7%, and 41.5%, respectively, compared to Na_2_SeO_3_ (Figures 10H-M), indicating that selenium nanoparticles restore immune homeostasis by limiting immune cell infiltration. Biocompatibility assessment showed no histopathological abnormalities in major organs (heart, liver, spleen, lung, kidney, and thyroid) and no significant alterations in blood biochemical markers (ALT, AST, BUN, CRE, etc.) in the Se@LNT group compared to controls (Figures S32-S33), confirming its superior safety profile relative to Na_2_SeO_3_ and supporting its potential as a low-toxicity, high-efficacy therapeutic strategy for TED. To evaluate the targeting capability of Se@LNT in orbital tissues, *in vivo* fluorescence imaging was performed from day 1 to day 4 post-injection. In the Se@LNT group, fluorescence intensity progressively increased, peaking on day 2, and declined by day 4, indicating time-dependent orbital accumulation (Figure S34A). In contrast, the Cy5- Na_2_SeO_3_ group showed only faint orbital fluorescence on day 2, suggesting limited tissue retention. Inductively coupled plasma mass spectrometry (ICP-MS) analysis further confirmed that selenium content in orbital tissues increased over time in the Se@LNT group and significantly declined by day 4 (Figure S34B), whereas the Na_2_SeO_3_ group exhibited markedly lower orbital selenium levels. *Ex vivo* imaging on day 3 revealed strong fluorescence in the liver and kidneys, and moderate signal in the thyroid for both groups, reflecting common metabolic pathways, although Se@LNT showed greater overall accumulation (Figure S34C). Through multidimensional regulation of immune homeostasis and effective tissue targeting, Se@LNT significantly reversed the pathological progression of TED in mice.

## Conclusion

This study proposes a novel therapeutic strategy for TED using Se@LNT. Se@LNT offers three major innovative advantages: (1) microenvironment-responsive delivery, in which ROS-sensitive Se-O coordination bonds enable the precise release of bioactive selenium under acidic and inflammatory conditions, facilitating its targeted accumulation in the mitochondria and endoplasmic reticulum of OFs; (2) metabolic conversion of Se@LNT into SeCys_2_, which is efficiently integrated into the active site of GPX1, restoring the GSH/GSSG ratio, reversing oxidative stress, reestablishing mitochondrial oxidative phosphorylation, and correcting glycolysis-dominated metabolic reprogramming; (3) suppression of excessive MAMs anchoring through GRP75 downregulation, thereby interrupting the calcium overload-mtDNA leakage-cGAS/STING cascade, and synergistically modulating redox and immune homeostasis. This work is the first to elucidate the molecular mechanism by which Se@LNT reverses adipose hyperplasia in TED via GPX1-mediated restoration of MAM function. It also demonstrates dual regulatory effects on immune rebalancing and inhibition of the PPARG/CEBPA-driven adipogenic network. Collectively, these findings not only introduce a selenium-based nanotherapeutic paradigm for TED, but also offer a new conceptual framework for targeting organelle interfaces to achieve integrated metabolic and immunological regulation.

## Supplementary Material

Supplementary experimental section, figures and tables.
