# Supplementary material for: GPX1-driven selenium nanoplatform reprograms MAMs-mediated organelle crosstalk to reverse inflammatory adipose expansion in thyroid eye disease
Source: Theranostics. 2025 Aug 30;15(18):9601–22. doi: 10.7150/thno.117582 (PMC12486268; doi:10.7150/thno.117582)
Supplement: Supplementary file 1 — Supplementary experimental section, figures and tables. [file thnov15p9601s1.pdf]

Supporting Information

GPX1-driven selenium nanoplatform reprograms MAMs-mediated organelle crosstalk to reverse inflammatory adipose expansion in thyroid eye disease

Yao Tan[1,2], Feng Zhang[1], Jiamin Cao[1], Lemeng Feng[1], Bingyu Xie[1], Limo Gao[1], Xiangdong Chen[3], Zuyun Yan[4,5], Wei Xiong[1]**

[1] Department of Ophthalmology, The Third Xiangya Hospital, Central South University, No. 138 Tongzipo Road, Yuelu District, Changsha City 410013, Hunan Province, China

[2] Postdoctoral Station of Clinical Medicine, The Third Xiangya Hospital, Central South University, Changsha City 410013, Hunan Province, China

[3] The First Affiliated Hospital of Hunan University of Chinese Medicine, Hunan Ophthalmic Disease (Traditional Chinese Medicine) Clinical Research Center, Changsha City 410000, Hunan Province, China

[4] Department of Spine Surgery, The Third Xiangya Hospital, Central South University, Changsha, Hunan, 410013, China

[5] Postdoctoral Station of Medical Aspects of Specific Environments, the Third Xiangya Hospital, Central South University, Changsha City 410013, China

*Correspondence:

Wei Xiong, Department of Ophthalmology, The Third Xiangya Hospital, Central South University, No. 138 Tongzipo Road, Yuelu District, Changsha City 410013, Hunan Province, China. E-mail: weixiong420@csu.edu.cn

Zuyun Yan, Department of Spine Surgery, The Third Xiangya Hospital, Central South University, Changsha, Hunan, 410013, China. E-mail: zyyancsu@163.com

Experimental Section

Materials

All cell culture media were purchased from Gibco (Rockville, MD, USA). Lentinan (LNT), ascorbic acid (VC), selenocystine ($SeCys_2$), selenomethionine (SeMet), methylselenocysteine (MeSeCys), Na_2SeO_3 (SeIV), Na_2SeO_4 (SeVI), DAPI, and other chemical reagents were acquired from Sigma-Aldrich (St. Louis, MO, USA). Cy5-NHS was obtained from Meilun Bio (Dalian, China). Female BALB/c mice (6-8 weeks old) were procured from Hunan Silaike Jingda Experimental Animal Co., Ltd. (Hunan Province, China). The animal experimental protocol was approved by the Animal Care and Use Committee of Central South University (Ethics Approval No. XMSB-2024-0215), and all procedures complied with the *Guide for the Care and Use of Laboratory Animals*.

Synthesis and characterization of Se@LNT

Se@LNT was synthesized following a previously reported method [1]. Briefly, LNT solution (2 mL, 1 mg/mL) was mixed with Na_2SeO_3 solution (2 mL, 10×10^{-3} M) under continuous stirring (400 rpm, 10 min). Subsequently, VC solution (2 mL, 40×10^{-3} M) was added dropwise, and the final volume was adjusted to 10 mL with Milli-Q water. The mixture was stirred at room temperature for 8 h, followed by dialysis against Milli-Q water (molecular weight cutoff: 6,000-8,000 kDa) for 24 h to remove unreacted reagents. The purified Se@LNT was stored at 4 °C.

Particle size and surface charge were characterized using dynamic light scattering (DLS) and zeta potential analysis, respectively. Morphological features were examined via transmission electron microscopy (TEM), and the chemical structure was analyzed by X-ray photoelectron spectroscopy (XPS). Stability assays were performed in PBS and DMEM medium, while the total antioxidant activity was evaluated using an ABTS (2,2′-azinobis[3-ethylbenzothiazoline-6-sulfonate]) radical scavenging assay.

Adipose tissue collection and ethical statement

Orbital adipose tissues were collected from 6 thyroid eye disease (TED) patients undergoing orbital decompression surgery and 6 control subjects undergoing strabismus or orbital procedures. For TED patients, inclusion criteria comprised individuals with enlarged extraocular muscles confirmed by CT/MRI scans and restricted ocular motility, while exclusion criteria included those who had received orbital radiotherapy or systemic steroid therapy within the preceding 3 months. Control subjects were enrolled based on diagnoses of concomitant

strabismus, cosmetic orbital decompression, enucleation due to trauma, or blepharoplasty, with exclusion of individuals presenting orbital inflammatory diseases, thyroid dysfunction, orbital infections, or prior intraorbital malignancies. Baseline characteristics of enrolled subjects are summarized in **Table S1**. All tissue collection protocols adhered to the principles of the *Declaration of Helsinki* and were approved by the Institutional Human Research Ethics Committee of the Third Xiangya Hospital of Central South University (Ethics Approval No. 2024-S109). Written informed consent was obtained from all participants.

Isolation and culture of primary orbital fibroblasts (OFs)

Primary OFs were isolated from TED and control orbital adipose tissues. Freshly excised tissues were immediately immersed in ice-cold saline, rinsed with PBS to remove superficial vasculature and blood cells, and minced into 1-3 mm^3 fragments. Tissue fragments were digested with collagenase type II (0.5 mg/mL, Sigma, C6885) in serum-free DMEM under constant agitation (37 °C, 50 min). The reaction was terminated by adding fetal bovine serum. The digestate was filtered through a 100 μm cell strainer, and the filtrate was centrifuged at 300 ×g for 5 min to pellet cells. Erythrocyte contamination was eliminated by incubating the pellet with erythrocyte lysis buffer (Beyotime, C3702; Shanghai, China) at room temperature for 10 min, followed by PBS washing. Cells were resuspended in complete culture medium and maintained under primary culture conditions (37 °C, 5% CO_2). Fresh medium was replenished after 48 h, and cells were passaged with trypsin upon reaching 70-80% confluence. Experiments utilized cells from passages 2-6 to ensure phenotypic stability.

GEO differential gene expression and GSEA analysis

The publicly available gene expression dataset GSE58331 from the GEO database was analyzed [2]. This dataset comprises transcriptomic profiles of orbital adipose tissues from 27 TED patients and 22 normal controls. Differential gene expression analysis was performed using the limma package (v3.40.6) in R. Genes with |log2FC| > 0.5 and adjusted $p < 0.05$ were considered differentially expressed. For Gene Set Enrichment Analysis (GSEA), the GSEA software (v3.0) was employed. Samples were stratified into high-expression (≥ 50%) and low-expression (< 50%) groups based on target gene expression levels. The c2.cp.kegg.v7.4.symbols.gmt subset from the Molecular Signatures Database was used to evaluate pathway enrichments. Spearman rank correlation analysis was conducted between MAMs core pathway genes and expressed genes from the GSE58331 dataset ($n = 49$).

Correlation coefficients (*r*) and significance (*p*-values) were calculated using the psych package (v2.4.12) in R (v4.2.1), with scatterplots generated via ggplot2 (v3.4.0).

Immunofluorescence analysis of paraffin-embedded sections

Orbital tissues were paraffin-embedded, and 4 μm-thick sections were prepared. Deparaffinization was performed using xylene, followed by rehydration in graded ethanol and PBS rinsing. Antigen retrieval was achieved by high-pressure heating in retrieval buffer (Beyotime, P0083). Sections were blocked with 0.01% Triton X-100-containing solution at room temperature for 1 h. Primary antibodies against GPX1 (Abcam, ab22604), STING (Proteintech, 19851-1-AP), CD3 (Abcam, ab16669), Ly6G (Abcam, ab25377), and IBA1 (Abcam, ab5076) were diluted (1:100) and incubated in a humidified chamber at 4 °C overnight. After warming to room temperature, sections were incubated with Alexa Fluor® 488-conjugated goat anti-rabbit IgG (1:100) for 4 h in the dark. Nuclei were counterstained with DAPI-containing antifade mounting medium (Invitrogen, P36962). Images were captured using a confocal microscope (Zeiss, LSM 980) at specified excitation wavelengths, and fluorescence intensity was quantified with ImageJ software.

Western blot analysis

OFs were pretreated with varying concentrations of Se@LNT for 2 h, followed by stimulation with H_2O_2 (200 μM) for 24 h or induction with adipogenic differentiation medium for 10 days. For phosphorylation analysis, cells were pretreated with Se@LNT (2 h) before exposure to H_2O_2 (200 μM, 1 h) or adipogenic medium (2 h). Total cellular proteins were extracted using RIPA lysis buffer (Beyotime, P0013C) and quantified via a BCA protein assay kit (Thermo Fisher, 23227). Equal protein amounts were resolved by SDS-PAGE and transferred onto PVDF membranes (Millipore, IPVH00010). After blocking with 5% (w/v) skimmed milk (1 h), membranes were incubated overnight at 4 °C with primary antibodies against GPX1 (Abcam, ab22604), β-actin (Cell Signaling Technology [CST], Danvers, MA, USA; #4967), FABP4 (CST, #2120), CEBPA (Abcam, ab317442), PLIN1 (Abcam, ab172907), PPARG (CST, #2435), BiP (CST, #3183), PERK (CST, #3192), p-PERK (Thr980; Invitrogen, MA5-15033), eIF-2α (CST, #9722), p-eIF-2α (Ser51; CST, #9721), ATF4 (CST, #11815), CHOP (CST, #5554), GRP75 (CST, #2816), VDAC1 (CST, #4866), IP3R1 (CST, #8568), STING (Proteintech, 19851-1-AP), p-STING (Ser365; CST, #72971), TBK1 (CST, #3504), p-TBK1 (Ser172; CST, #5483), IRF3 (CST, #4302), p-IRF3 (Ser396; CST, #29047), NF-κB (CST, #8242), and p-NF-κB (Ser536; CST, #3033). HRP-conjugated secondary antibodies

(CST, #7074S) were applied (1 h, room temperature), and protein bands were visualized using an ECL reagent (Thermo Fisher, 34577).

Glutathione peroxidase (GSH-Px) activity assay

Tissue and cell samples were processed to measure GSH-Px activity using a commercial assay kit (Beyotime, S0056). Tissue specimens were homogenized in saline according to the manufacturer's protocol, while primary cells were lysed via standard procedures to obtain supernatants. Protein concentrations were normalized using the BCA method, and enzymatic activity was quantified based on kinetic measurements as outlined in the kit instructions.

GSH/GSSG ratio measurement

The reduced (GSH) and oxidized (GSSG) glutathione levels were quantified using a Total Glutathione Assay Kit (Beyotime, S0053). Tissue and cell lysates were processed according to the manufacturer's protocol, including protein removal and standard curve preparation. Absorbance was dynamically monitored at 412 nm. Total glutathione and GSSG concentrations were calculated based on the standard curve, and the GSH/GSSG ratio was derived using the formula: (Total Glutathione - 2 × GSSG) / GSSG.

Detection of lipid peroxidation products

Malondialdehyde (MDA) levels, a marker of lipid peroxidation, were measured using a thiobarbituric acid (TBA) assay kit (Beyotime, S0131S). Tissue and cell lysates were reacted with TBA under boiling water bath conditions as per the manufacturer's instructions, and absorbance was recorded at 532 nm.

Flow cytometric characterization of OFs

OFs were stained with the following fluorochrome-conjugated antibodies: APC anti-human CD44 (BD Biosciences, 559942), PE-Cy7 anti-human CD45 (BD Biosciences, 560915), APC anti-human CD90 (BD Biosciences, 559869), FITC anti-human CD73 (BD Biosciences, 561254), PE anti-human CD105 (BD Biosciences, 560839), FITC anti-human HLA-DR (BD Biosciences, 560944), APC anti-human CD19 (BD Biosciences, 560727), and PE anti-human CD11b (BD Biosciences, 555388). Cells were incubated with antibodies (1:100 dilution) at 4 °C for 30 min under light-protected conditions. Flow cytometric analysis was performed using a BD Accuri™ C6 Plus flow cytometer (BD Biosciences). Data were analyzed with FlowJo software (v10.8.1).

Mitochondrial membrane potential (MMP) assay

MMP was dynamically assessed using the JC-1 probe (10 μM, Beyotime, C2003). Cells were washed with PBS and incubated with JC-1 in serum-free medium (37 °C, 30 min, dark). Fluorescence signals were captured via confocal microscopy (excitation/emission: 488/530 nm for green monomers; 543/590 nm for red aggregates). The MMP levels were calculated as the ratio of red-to-green fluorescence intensity (red fluorescence intensity / green fluorescence intensity). All images were acquired using the EVOS M7000 Imaging System (Invitrogen).

Mitochondrial reactive oxygen species (mtROS) detection

Mitochondrial ROS levels were monitored using MitoSOX Red (5 μM, Beyotime, S0061S) and MitoTracker Green (100 nM, Beyotime, C1048). Cells were washed with PBS and co-stained with both probes in medium containing 2% FBS (37 °C, 30 min, dark). Nuclei were counterstained with DAPI (1 μg/mL, 10 min). Confocal microscopy was employed to visualize mitochondrial ROS distribution and morphology. Flow cytometry (BD Accuri™ C6 Plus) was concurrently utilized for quantitative mtROS analysis. Fluorescence imaging was performed using the EVOS M7000 Imaging System (Invitrogen).

Immunofluorescence (IF) staining

Cells or frozen sections were fixed with 4% (w/v) paraformaldehyde, permeabilized with 0.2% (v/v) Triton X-100 for 30 min, and blocked with 5% (w/v) bovine serum albumin (BSA) at room temperature for 2 h. Samples were incubated overnight at 4 °C with primary antibodies against GPX1 (Abcam, ab22604), Calnexin (CST, #2433), TOMM20 (CST, #42406), P4HB (CST, #3501), dsDNA (Santa Cruz Biotechnology, sc-58749), and 8-OHdG (Santa Cruz Biotechnology, sc-66036). After washing, sections were incubated with fluorophore-conjugated secondary antibodies (1:100 dilution) at room temperature for 2 h, followed by DAPI counterstaining (10 min). Images were acquired using a confocal laser-scanning microscope (Zeiss, LSM 980).

Cell viability assay (CCK-8)

OFs in the logarithmic growth phase were seeded into 96-well plates (5×10^3 cells/well). Peripheral wells were filled with PBS to minimize evaporation, and cells were allowed to adhere at 37 °C for 12 h. Cells were treated with drug solutions at varying concentrations (0, 1.25, 2.5, 5, 10, 20, and 40 mM) or complete medium (control group), with each concentration tested in 3-5 replicate wells. After 24 h of incubation, the medium was replaced with fresh medium containing 10% CCK-8 reagent (MedChem Express, HY-K0301-500T, 110 μL/well), followed by incubation at 37 °C in the dark for 1 h. Absorbance at 450 nm was measured using

a microplate reader, and relative cell viability was calculated as a percentage of the control group.

Selenium speciation analysis by HPLC-ICP-MS

Selenium speciation following Se@LNT treatment was analyzed using high-performance liquid chromatography coupled with inductively coupled plasma mass spectrometry (HPLC-ICP-MS), as previously described [1]. Briefly, primary orbital fibroblasts were treated with Se@LNT (10 μM) for 3, 6, and 12 hours. At each time point, cells were harvested, washed with PBS, and lysed using a digestion buffer containing proteinase K and trypsin. The lysates were centrifuged, and the supernatants were collected and filtered through 0.22 μm membranes prior to analysis. Chromatographic separation of selenium species was performed using a reversed-phase C18 column. The mobile phase consisted of 10 mM citric acid solution (pH 4.5), delivered at a flow rate of 0.8 mL/min. Selenium species were detected using an ICP-MS system operated in standard mode with appropriate Se isotopes for quantitative analysis. Retention times were compared with standard references for $SeCys_2$, MeSeCys, Se(IV), SeMet, and Se(VI) to identify intracellular selenium metabolites.

Intracellular localization of nanoparticles

Se@LNT nanoparticles (10 mg) and Cy5-NHS ester (0.5 mg) were dissolved in PBS (5 mL, 4 °C) and stirred under light-protected conditions for 24 h. The mixture was dialyzed against PBS using a 3.5 kDa molecular weight cutoff membrane (4 °C, 72 h) to remove unbound Cy5. Primary OFs were seeded into 35 mm confocal dishes (3×10^4 cells/mL) and cultured for 24 h (37 °C, 5% CO_2). Cells were treated with Cy5-Se@LNT (50 μg/mL) for 2 h, fixed with 4% paraformaldehyde (15 min), permeabilized with 0.1% Triton X-100 (10 min), and washed with PBS. F-actin was stained with Alexa Fluor 488-phalloidin (Abcam, ab206277, 30 min, dark), followed by DAPI nuclear staining (5 min). Images were acquired using a CLSM after mounting with antifade reagent.

Quantitative real-time PCR (qRT-PCR)

Total RNA was extracted from primary orbital fibroblasts using TRIzol reagent (Thermo Fisher Scientific, 15596026CN) and purified via chloroform-isopropanol precipitation. RNA purity was confirmed by spectrophotometric analysis (A260/A280 ≥ 1.8). cDNA synthesis was performed from 1 μg RNA using the EasyScript® One-Step gDNA Removal and cDNA Synthesis Kit (TransGen Biotech, AE311). qPCR amplification was carried out on a StepOne Plus system (Thermo Fisher Scientific) with PerfectStart® Green SuperMix (TransGen Biotech,

AQ601). Primer specificity was validated by melt curve analysis. Target gene expression levels were normalized to β-actin and calculated using the $2^{-\Delta\Delta Ct}$ method. Primer sequences for selenium-related genes were referenced from previous work [3], with sequences provided in **Table S2**.

Intracellular reactive oxygen species (ROS) detection

ROS levels were measured using the H2DCF-DA probe (MedChem Express, HY-D0940). After PBS washing, cells were incubated with H2DCF-DA (10 μM, 37 °C, 30 min, dark) and counterstained with DAPI (10 min). Fluorescence signals were visualized via CLSM and quantified by flow cytometry (BD Accuri™ C6 Plus, BD Biosciences).

Lipid peroxidation assay

Lipid peroxidation was assessed using the C11-BODIPY 581/591 probe (Thermo Fisher, D3861). Cells were incubated with C11-BODIPY (5 μM, 30 min, 37 °C, dark) in medium containing 2% FBS. Nuclei were counterstained with DAPI, and dual-channel fluorescence (oxidized and non-oxidized states) was captured by CLSM. The relative lipid peroxidation (LPO) intensity was calculated as: Relative LPO intensity= Oxidized fluorescence intensity / Non-oxidized fluorescence intensity. Fluorescence intensity per cell membrane region was quantified using ImageJ.

Lactate dehydrogenase (LDH) release assay

LDH release was quantified using a commercial kit (Beyotime, C0016). Cell supernatants were collected and incubated with LDH detection reagent (30 min, dark). Absorbance was measured at 490 nm using a microplate reader.

Lactate concentration measurement

Intracellular L-lactate levels were quantified using a commercial assay kit (Abcam, ab65330). Primary orbital fibroblasts were lysed and centrifuged, and the resulting supernatants were deproteinized according to the manufacturer's protocol. Absorbance was measured at 450 nm, and data were normalized to total protein concentration.

Transmission electron microscopy analysis

OFs and mouse orbital adipose tissues were fixed with primary electron microscopy fixative, post-fixed with 2% osmium tetroxide, and dehydrated through an ethanol gradient. Samples were embedded in Epok 812 resin and sectioned into 70 nm ultrathin slices using an ultramicrotome. Sections were double-stained with uranyl acetate and lead citrate, and cellular ultrastructure was examined using a transmission electron microscope (JEOL, JEM-1400Flash).

Mouse samples were dissected from the central orbital region, and fibroblast-rich areas were selected for ultrastructural assessment of mitochondria, endoplasmic reticulum, and MAMs.

Seahorse extracellular flux analysis

Mitochondrial and glycolytic functions of orbital fibroblasts were evaluated using the Seahorse XF96 Analyzer (Agilent) according to the manufacturer's protocols. Cells were seeded in XF96 plates and incubated overnight. For OCR measurements, cells were incubated in base medium (pH 7.4) supplemented with 10 mM glucose, 2 mM glutamine, and 1 mM sodium pyruvate; for ECAR, glucose-free medium with 2 mM glutamine was used. Plates were equilibrated at 37 °C in a non-CO_2 incubator for 1 h.

The Mito Stress Test involved sequential injections of oligomycin (1 μM), FCCP (1 μM), and rotenone/antimycin A (0.5 μM each) to assess mitochondrial function. The Glycolysis Stress Test included injections of glucose (10 mM), oligomycin (1 μM), and 2-DG (50 mM) to evaluate glycolytic parameters. Data were normalized to cell number and analyzed using Wave software (Agilent) and GraphPad Prism 9.0.

Adipogenic differentiation of fibroblasts

Primary OFs were seeded in 6-well plates and cultured to 100% confluence. Adipogenic differentiation was induced using a commercial adipogenesis induction kit (Procell, PD-006), with medium replaced every 3 days for 10 days.

Oil Red O staining

To assess lipid droplet formation, differentiated OFs were fixed with 4% paraformaldehyde (20 min), washed with PBS, and stained with Oil Red O solution (Sigma, O0625) at room temperature for 30 min in the dark. After rinsing to remove unbound dye, images of lipid droplets were captured using an inverted microscope. Lipid droplet area was quantified using ImageJ software.

AdipoRed assay

Differentiated OFs were washed with PBS to remove residual medium and incubated with AdipoRed reagent (Lonza, PT-7009) diluted 1:200 in PBS (37 °C, 10 min, dark). Fluorescent images were acquired using a fluorescence microscope. The proportion of AdipoRed-positive cells per field was analyzed by thresholding fluorescence intensity in ImageJ.

Triglyceride content measurement

Cellular triglyceride levels were quantified using the Amplex® Red Triglyceride Assay Kit (Beyotime, S0219S). Lipids were extracted from adipogenically differentiated orbital

fibroblasts via isopropanol homogenization, diluted 5-fold according to the manufacturer's protocol, and incubated with the triglyceride detection working solution in the dark. Fluorescence intensity was measured using a fluorescence microplate reader (excitation/emission: 530/590 nm).

Mitochondrial permeability transition pore (MPTP) assay

Mitochondrial membrane permeability was assessed using the MPTP Assay Kit (Beyotime, C2009S). Primary orbital fibroblasts were incubated with Calcein AM (2 μM) and $CoCl_2$ (250 μM) in quenching buffer (30 min, 37 °C, dark). Cells were washed and further incubated in pre-warmed DMEM containing 10% FBS (40 min, 37 °C). Fluorescence images were captured using a fluorescence microscope, and mitochondrial Calcein intensity was quantified using ImageJ.

siRNA transfection

To assess whether the regulatory effect of Se@LNT on GRP75 expression is dependent on GPX1, siRNA-mediated gene silencing was performed. Orbital fibroblasts were transfected with 50 nM GPX1-targeting siRNA (sense: GGUACUACUUAUCGAGAAUTT; antisense: AUUCUCGAUAAGUAGUACCTT) or negative control siRNA (GenePharma, China) using Lipofectamine RNAiMAX (Thermo Fisher Scientific) according to the manufacturer's protocol. After 48 h of transfection, cells were treated with H_2O_2 (200 μM, 24 h) with or without Se@LNT (10 μM, pretreated for 2 h). Cells were then harvested for Western blot analysis to evaluate the expression of GPX1 and GRP75.

Cytosolic mtDNA detection

Cytosolic fractions were isolated using a Mitochondrial/Cytosol Fractionation Kit (Beyotime, C3601). Mitochondrial debris was removed by high-speed centrifugation (12,000 ×g, 10 min), and cytosolic DNA was extracted using a QIAamp DNA Mini Kit (Qiagen, 51304). QPCR was performed to measure the abundance of mitochondrial DNA (mtDNA) genes *MTND1* and *MTND2*, normalized to nuclear genomic DNA (gDNA) via the ΔCt method.

p65 nuclear translocation assay

Nuclear translocation of p65 was assessed using an NF-κB Activation Detection Kit (Beyotime, SN368). Primary orbital fibroblasts were fixed with 4% paraformaldehyde (PFA), permeabilized, and incubated with rabbit anti-human p65 polyclonal antibody (1:100) at room temperature for 1 h. Cy3-conjugated secondary antibodies were applied under light-protected conditions, followed by DAPI counterstaining. Images were captured using a confocal

microscope, and the nuclear/cytoplasmic fluorescence intensity ratio was quantified using ImageJ.

ELISA

Soluble ICAM-1, interleukin-6 (IL-6), and tumor necrosis factor-α (TNF-α) levels in cell culture supernatants were measured using Human ICAM-1/CD54 (R&D Systems, DY720), Human IL-6 (R&D Systems, Q6000B), and Human TNF-α (R&D Systems, DTA00D) ELISA kits, respectively. Intracellular 2'3'-cGAMP levels were quantified with a 2'3'-Cyclic GAMP ELISA Kit (Invitrogen, EIAGAMP). Serum concentrations of T4, TSH, and TRAb in mice were determined using Mouse T4 (Ruixin Bio, RXJ202844M), Mouse TSH (Ruixin Bio, RX203002M), and Mouse TRAb (Ruixin Bio, RX200938M) ELISA kits. In addition, mouse serum levels of IL-6, TNF-α, and ICAM-1 were measured using Mouse IL-6 ELISA Kit (Proteintech, KE10007), Mouse TNF-α ELISA Kit (Proteintech, KE10002), and Mouse ICAM-1 ELISA Kit (Proteintech, KE10129), respectively. All procedures strictly adhered to the manufacturer's protocols.

Transcriptome sequencing and analysis

Total RNA was extracted from primary orbital fibroblasts using TRIzol reagent (Thermo Fisher Scientific, 15596026), with purity confirmed by A260/A280 ratios ≥ 1.8 (NanoDrop 2000, Thermo Fisher Scientific) and RNA integrity (RIN > 6.5) assessed via Agilent 5300. Library preparation and sequencing were commercially processed by Shanghai Majorbio Bio-pharm Technology Co., Ltd. on an Illumina NovaSeq X Plus platform. Briefly, mRNA was enriched using Oligo(dT)-attached magnetic beads, fragmented, and reverse-transcribed into double-stranded cDNA. Illumina adapters were ligated, and PCR amplification was performed to generate paired-end libraries (150 bp). Raw sequencing data were filtered using fastp (v0.23.4) to remove low-quality reads and adapter contamination. High-quality clean reads were aligned to the human reference genome GRCh38 using HISAT2 (v2.2.1), and gene expression abundance was quantified via RSEM (v1.3.3). Differentially expressed genes (DEGs) were identified using DESeq2 (v1.38.3) with thresholds of $|\log2FC| \geq 1$ and false discovery rate (FDR) < 0.05. Functional annotation and KEGG pathway enrichment were performed using KOBAS (v3.0), with significance defined as $p < 0.05$ after Benjamini-Hochberg correction. Three-dimensional principal component analysis (PCA) was conducted using the stats package (v4.2.1) in R. Hierarchical clustering was performed based on Euclidean

distance matrices of TPM-normalized expression values. Gene co-expression patterns were analyzed via k-means clustering to identify functional modules.

Establishment of TED mouse model and selenium intervention

Female BALB/c mice (6-8 weeks old) were randomized into four groups (n = 6 per group): control, TED model, Se@LNT-treated, and Na_2SeO_3-treated. The TED model was induced via intramuscular injection of TSHR-encoding adenovirus (Ori-bio, PSB1317; 50 μL, 1.5×10^{10} viral particles [VP]/mL in PBS) into the quadriceps every 3 weeks for 12 weeks. Control mice received equivalent doses of empty adenovirus (Ori-bio, PMT407). The Se@LNT group received intraperitoneal injections of selenium nanoparticles (0.5 mg Se/kg) every 3 days from week 0, while the Na_2SeO_3 group was supplemented with sodium selenite (0.3 mg/L) via drinking water until study termination. Body weight, eyelid swelling, and conjunctival hyperemia were monitored weekly. At week 15, mice were euthanized under 1% isoflurane anesthesia. Serum levels of T4, TSH, and TRAb were measured by ELISA, and orbital adipose/fibrous tissues were fixed in 4% paraformaldehyde for histopathological analysis.

Magnetic resonance imaging (MRI)

On the day of sacrifice, orbital and extraocular muscle imaging was performed using a 7.0T MRI scanner (Bruker BioSpin) under isoflurane anesthesia. T2-weighted images were acquired as previously described, with acquisition parameters detailed in the referenced protocol [4].

Hematoxylin-Eosin (HE) staining

Thyroid, orbital tissues, and murine visceral organs (heart, liver, spleen, lung, kidney) were fixed in 4% neutral-buffered paraformaldehyde (24 h), paraffin-embedded, and sectioned at 4 μm thickness. Sections were deparaffinized in xylene I/II (10 min each), rehydrated through graded ethanol, and stained with hematoxylin (7 min). After rinsing under running water (10 min), sections were differentiated in 1% acid alcohol (1 min), rinsed again (10 min) for nuclear bluing, and counterstained with 0.5% eosin aqueous solution (2 min). Subsequently, sections were dehydrated in graded ethanol, cleared in xylene, and mounted with neutral resin for histological evaluation under a light microscope.

Oil Red O staining of orbital tissues

Orbital adipose tissues were embedded in OCT compound, cryosectioned, and air-dried. Sections were rinsed with distilled water (20 s), pretreated with 60% isopropanol (20 s) to enhance dye permeability, and stained with Oil Red O working solution (15 min, protected from

light) to visualize neutral lipid droplets. Non-specific background staining was removed by brief differentiation in 60% isopropanol (2 s). Nuclei were counterstained with Mayer's hematoxylin (2 min), rinsed with distilled water, and mounted with glycerin gelatin. Lipid droplet distribution was analyzed using light microscopy.

Blood biochemistry and hematological safety assessment

Whole blood samples collected from mice were aliquoted into heparin sodium-coated and clot activator tubes, followed by centrifugation (3000 ×g, 15 min) to separate serum and plasma. Serum levels of alanine aminotransferase (ALT), aspartate aminotransferase (AST), blood urea nitrogen (BUN), and creatinine were analyzed using an automated biochemical analyzer (Hitachi 7180, Tokyo, Japan). Hematological parameters, including red blood cell count, hemoglobin concentration, total leukocyte count, lymphocyte/monocyte absolute values, and platelet count, were quantified with an automated hematology analyzer (Mindray BC-5390CRP, Shenzhen, China).

***In vivo* biodistribution imaging**

To compare the biodistribution of Se@LNT with free selenium, Cy5-labeled Na_2SeO_3 (Cy5-Na_2SeO_3) was prepared as follows: 10 mg Na_2SeO_3 and 0.5 mg Cy5-NHS ester were dissolved in 5 mL PBS (4 °C) and stirred under light-protected conditions for 24 h. The solution was dialyzed against PBS using a 3.5 kDa cutoff membrane (4 °C, 72 h) to remove unbound Cy5.

TED mice were intraperitoneally injected with either Cy5-Se@LNT or Cy5-Na_2SeO_3 at a selenium dose of 0.5 mg/kg. Whole-body fluorescence imaging was performed at days 1, 2, 3, and 4 post-injection using an IVIS Spectrum system (PerkinElmer, Waltham, MA, USA). At Day 3, mice were sacrificed, and major organs (heart, liver, spleen, lung, kidney, thyroid) were excised for ex vivo imaging. Targeted accumulation was quantified via fluorescence intensity analysis. Selenium content in orbital tissues was measured by inductively coupled plasma mass spectrometry (ICP-MS; Thermo Fisher Scientific, iCAP RQ).

Statistical Analysis

All statistical analyses and graphical representations were conducted using GraphPad Prism 8 (GraphPad Software, San Diego, CA, USA) and Origin 2023 (OriginLab, Northampton, MA, USA). Data are expressed as mean ± standard error (SE). Comparisons among multiple groups were performed using one-way analysis of variance (ANOVA), while pairwise

comparisons were assessed via Student's *t*-test. A probability value of $p < 0.05$ was considered statistically significant. Significance levels are denoted as follows: *$p < 0.05$, **$p < 0.01$, and ***$p < 0.001$; ns indicates no significant difference.

Supplementary Materials

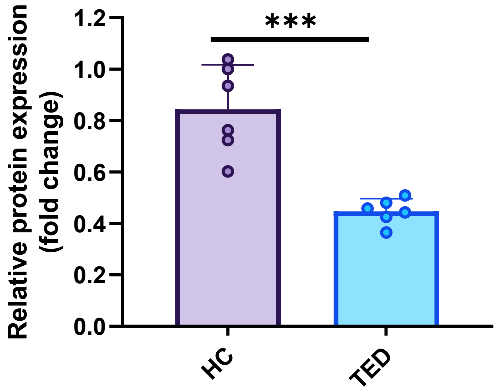

Figure S1. Quantitative analysis of GPX1 protein expression in orbital adipose tissues. (**$p < 0.01$; mean ± SE, $n = 6$)

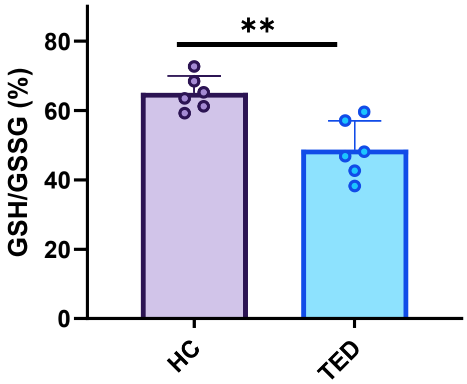

Figure S2. Measurement of GSH/GSSG ratio in orbital adipose tissues. (**$p < 0.01$; mean ± SE, $n = 6$)

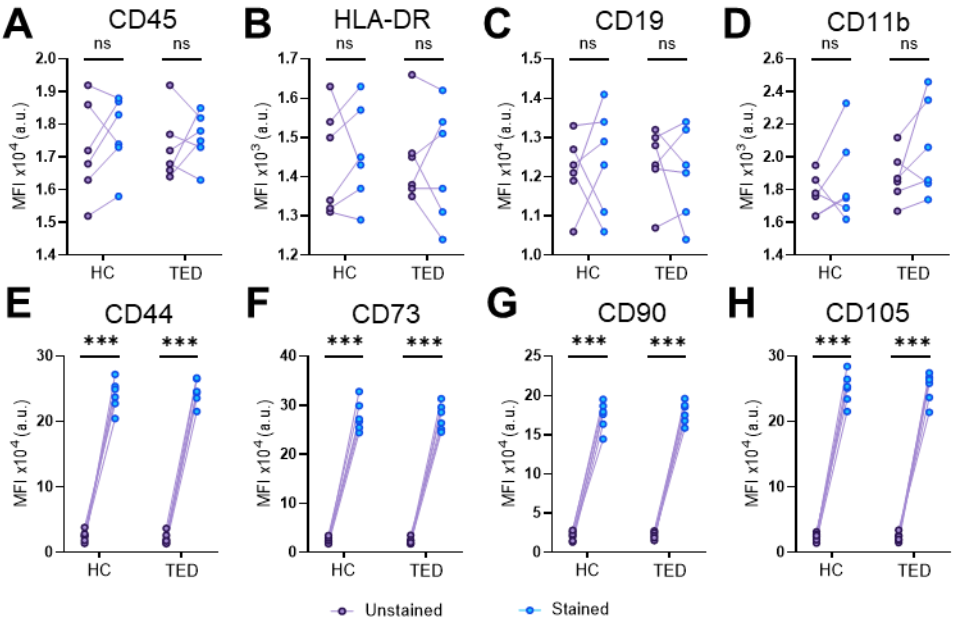

Figure S3. Flow cytometric quantification of immune and mesenchymal markers in primary orbital fibroblasts from healthy controls (HC) and TED patients. Representative bar graphs show the mean fluorescence intensity (MFI) of each marker: (A) CD45, (B) HLA-DR, (C) CD19, (D) CD11b, (E) CD44, (F) CD73, (G) CD90, and (H) CD105. (ns, no significance; ***$p < 0.001$; mean ± SE, n = 6)

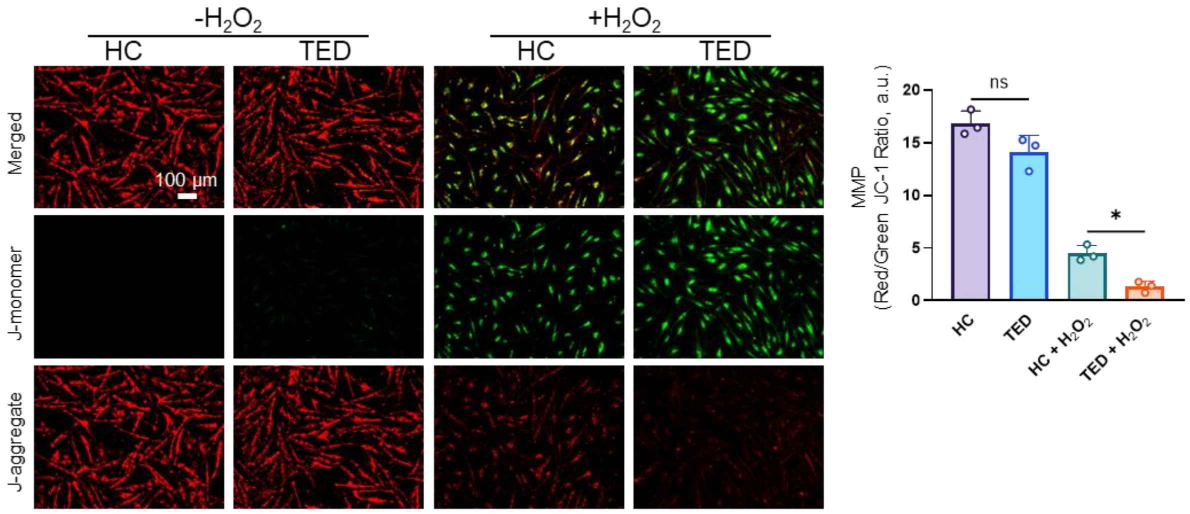

Figure S4. JC-1 staining and quantification of mitochondrial membrane potential in healthy and TED-derived OFs under baseline and oxidative stress conditions. Scale bar: 100 µm. (ns, no significance; *$p < 0.05$; mean ± SE, n = 3)

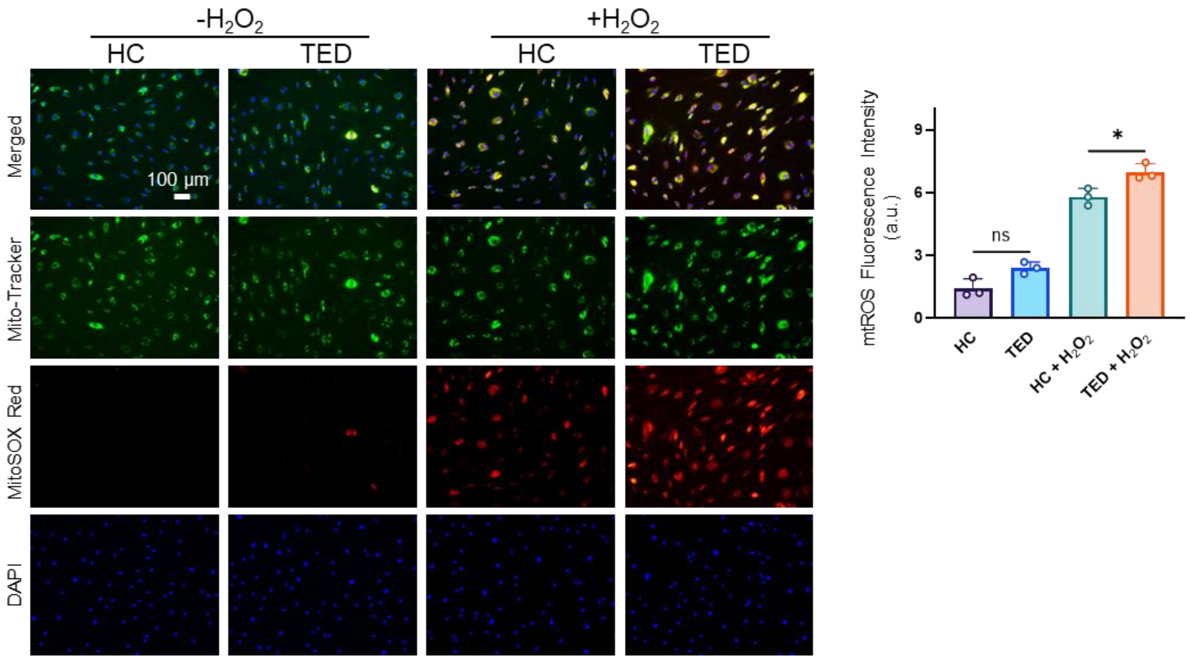

Figure S5. MitoSOX Red staining and quantification of mitochondrial reactive oxygen species (mtROS) in healthy and TED-derived OFs under baseline and oxidative stress conditions. Scale bar: 100 µm. (ns, no significance; **p* < 0.05; mean ± SE, n = 3)

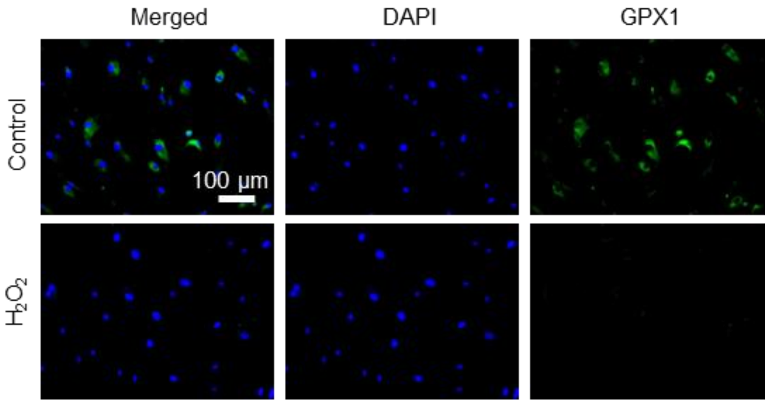

Figure S6. Immunofluorescence detection of GPX1 expression in TED-derived OFs induced by H_2O_2. Scale bar: 100 µm.

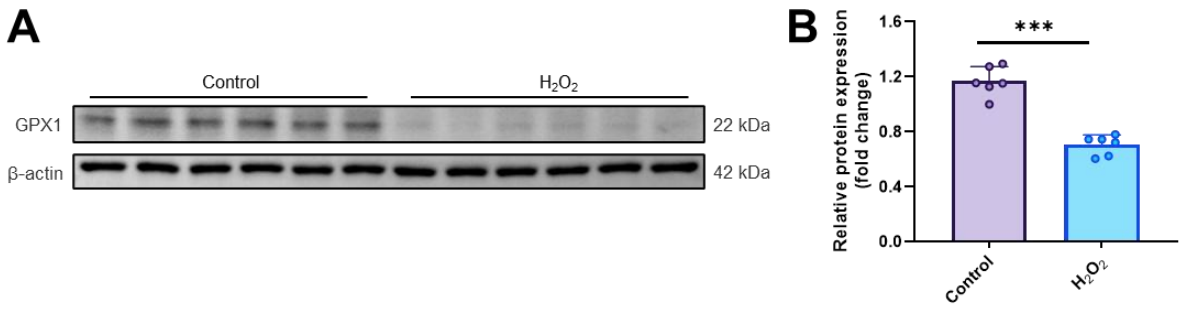

Figure S7. GPX1 protein expression in TED-OFs post H_2O_2 stimulation. (A) Western blot analysis of GPX1 protein levels. (B) Quantification of GPX1 protein expression. (***$p < 0.001$; mean ± SE, n = 6)

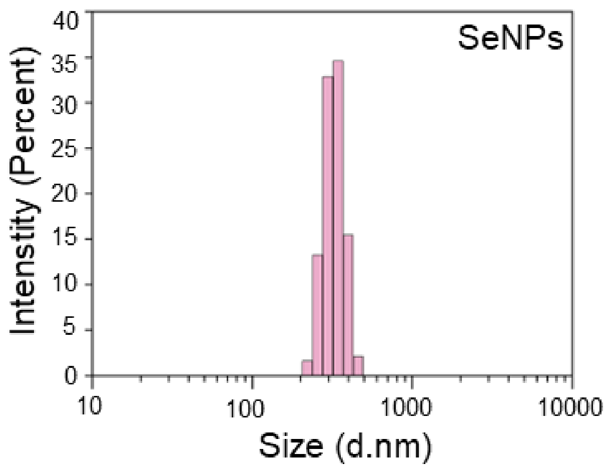

Figure S8. Hydrodynamic size distribution of SeNPs.

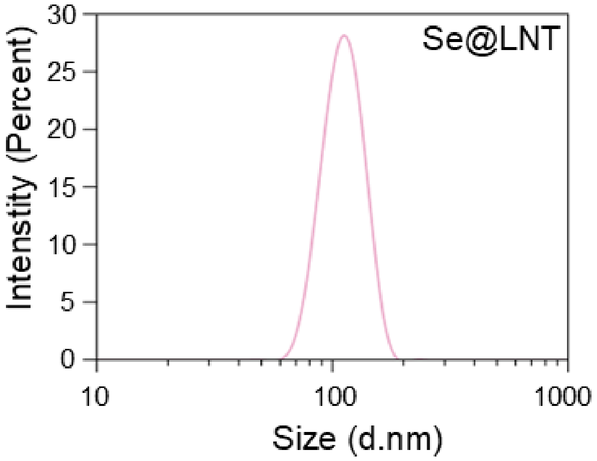

Figure S9. Size distribution fitting curve of Se@LNT nanoparticles.

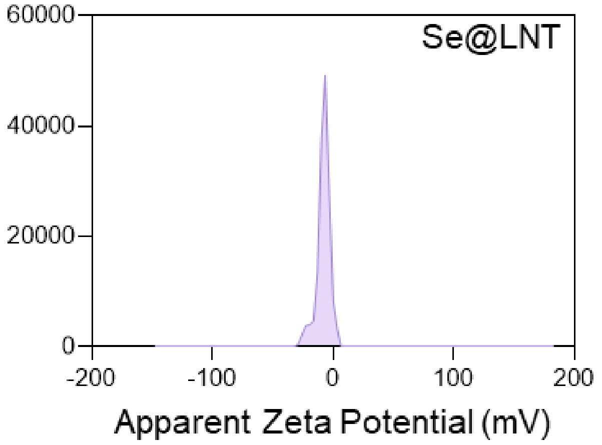

Figure S10. Zeta potential distribution profile of Se@LNT nanoparticles.

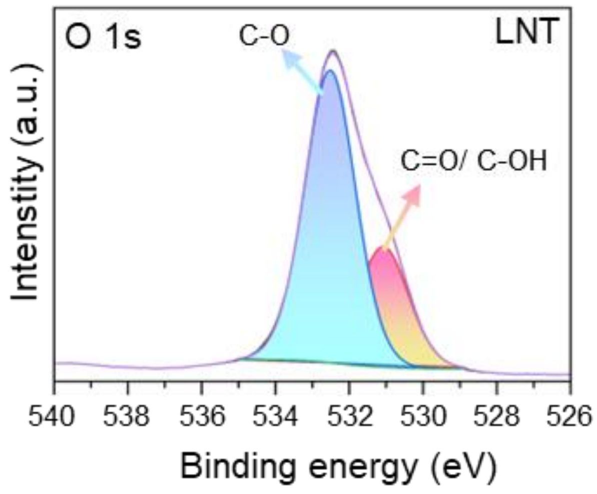

Figure S11. XPS spectrum of O 1s binding energy in lentinan (LNT).

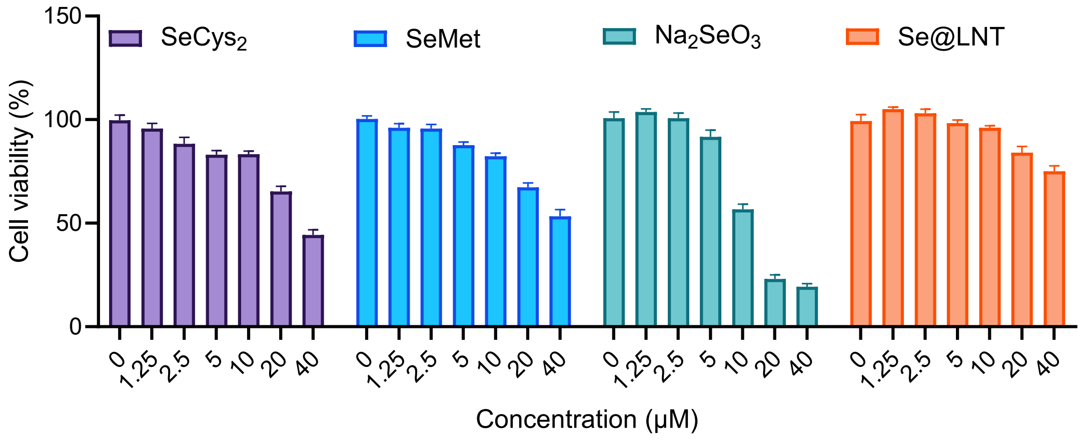

Figure S12. Viability of OFs cells treated with various selenium-containing agents ($SeCys_2$, SeMet, Na_2SeO_3, Se@LNT) at concentrations ranging from 0 to 40 μM for 48 hours. (mean ± SE, n = 3)

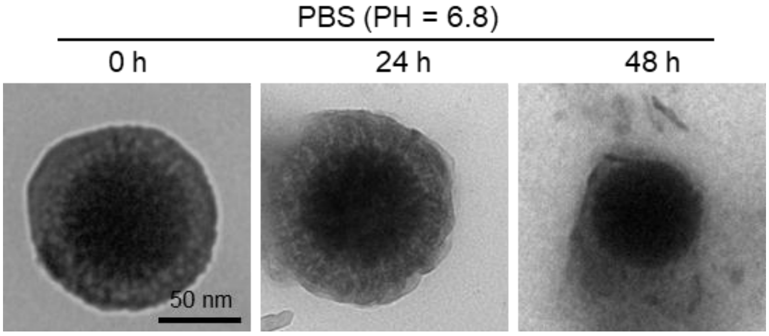

Figure S13. TEM images of Se@LNT after incubation in PBS (pH 6.8) without H_2O_2 for 24 h and 48 h. Scale bars: 50 nm.

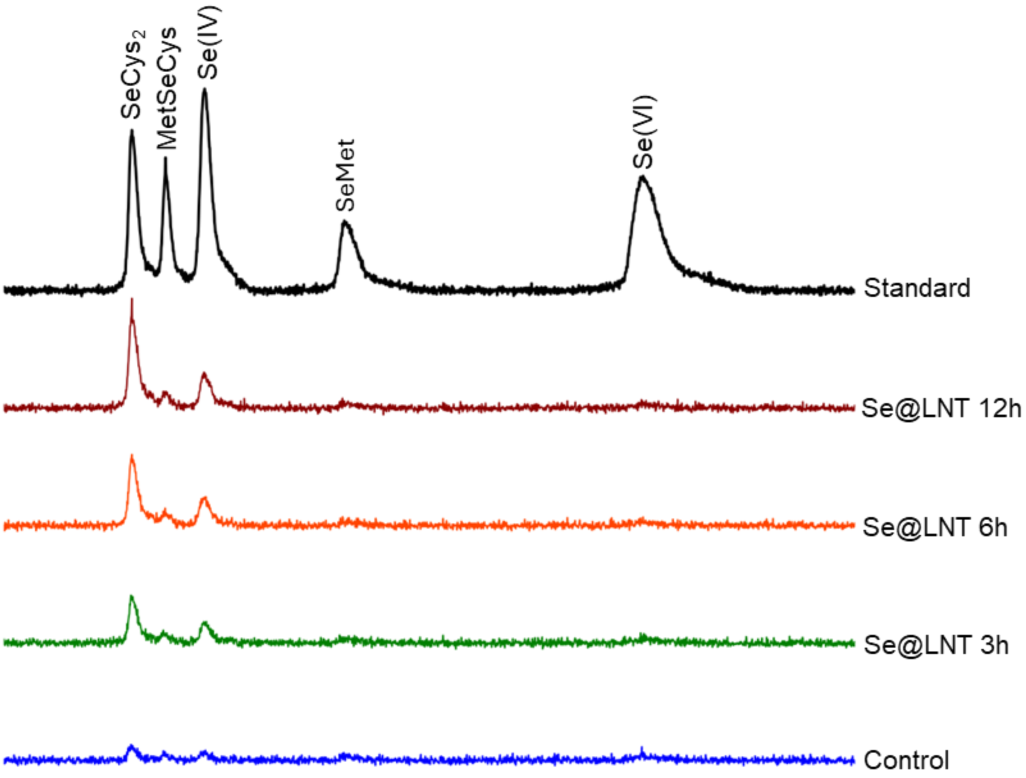

Figure S14. Representative HPLC-ICP-MS chromatograms showing selenium species in orbital fibroblasts after treatment with Se@LNT at different time points. Standard reference (top) displays distinct peaks corresponding to $SeCys_2$, MeSeCys, Se(IV), SeMet, and Se(VI).

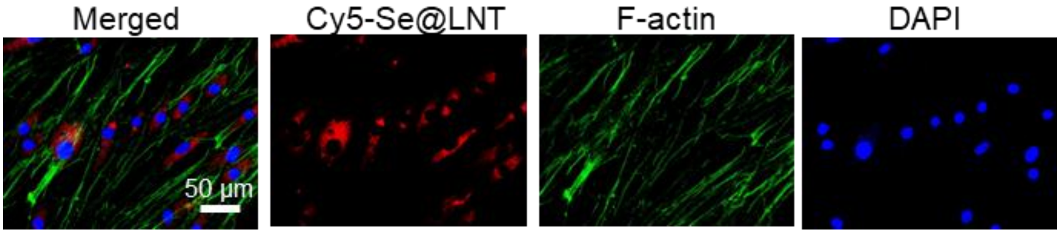

Figure S15. Intracellular localization of Se@LNT nanoparticles in OFs. Scale bar: 50 μm.

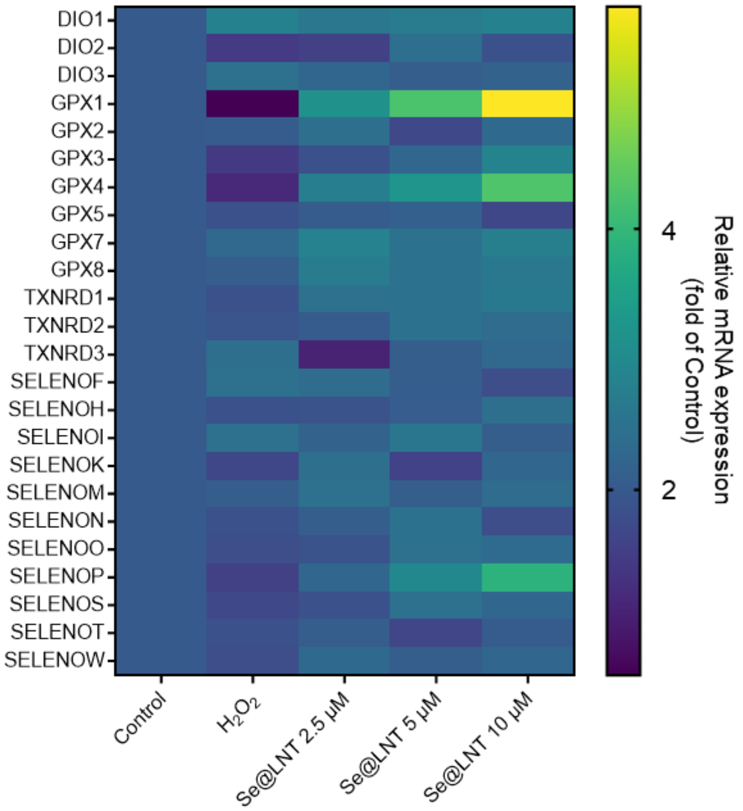

Figure S16. mRNA expression of selenoproteins in OFs under different treatments. (mean ± SE, n = 3)

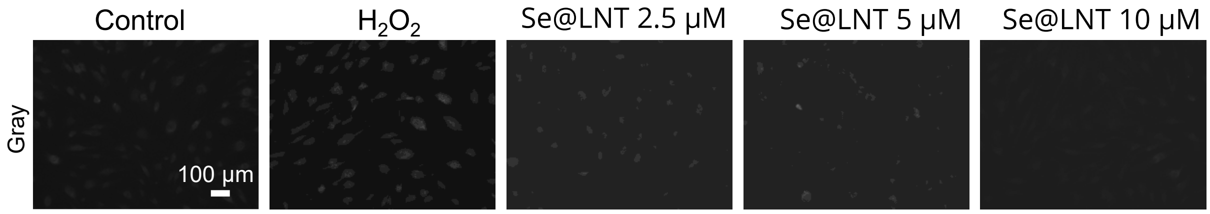

Figure S17. Representative grayscale images confirming the spindle-shaped morphology of primary OFs used in mtROS detection (corresponding to Figure 4C). Scale bar: 100 μm.

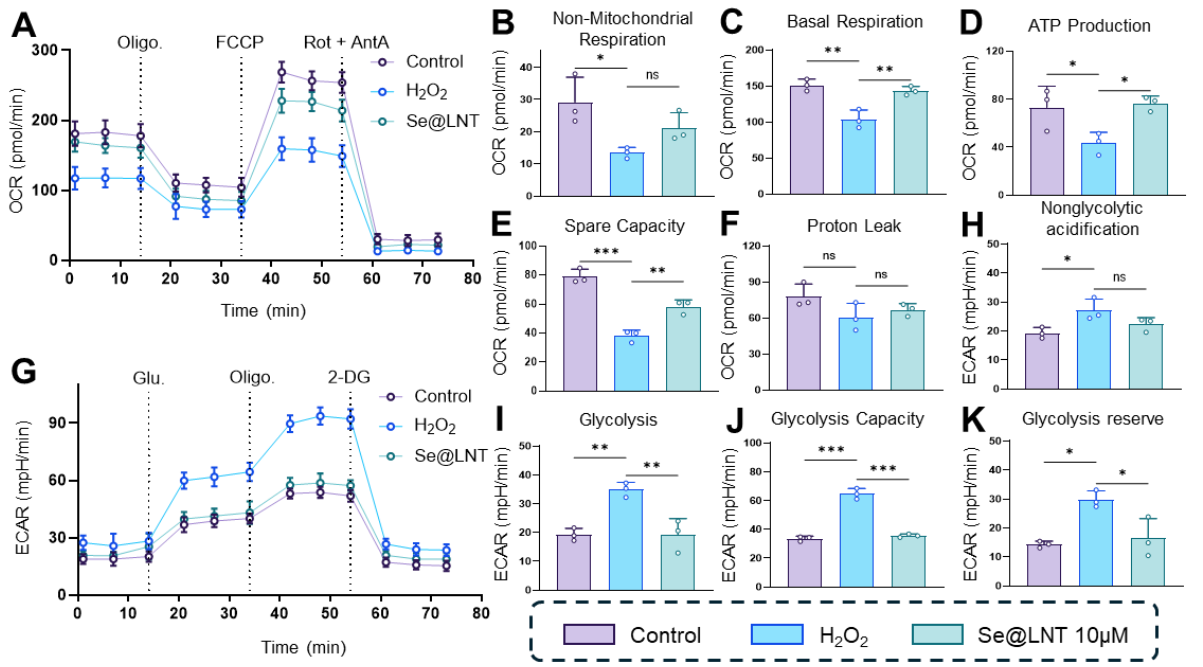

Figure S18. Seahorse XF96 analysis of mitochondrial and glycolytic function in OFs following H_2O_2 stimulation and Se@LNT treatment. (A) Oxygen consumption rate (OCR) traces over time reflecting overall mitochondrial respiration. (B-F) Quantitative analysis of mitochondrial parameters derived from the OCR profile, including (B) non-mitochondrial respiration, (C) basal respiration, (D) ATP production, (E) spare respiratory capacity, and (F) proton leak. (G) Extracellular acidification rate (ECAR) traces over time indicating glycolytic activity. (H-K) Quantification of glycolysis-related parameters derived from the ECAR curve, including (H) nonglycolytic acidification, (I) glycolysis, (J) glycolytic capacity, and (K) glycolytic reserve. (ns, no significance; *$p < 0.05$; **$p < 0.01$; ***$p < 0.001$; mean ± SE, $n = 3$)

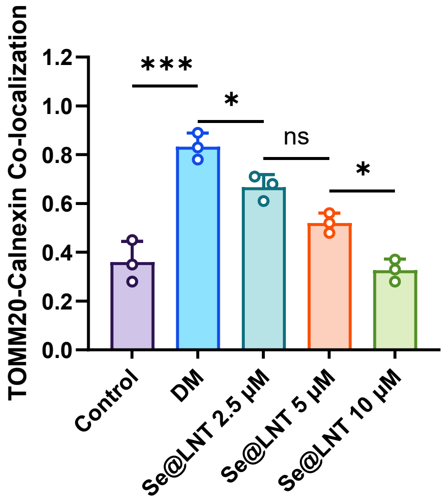

Figure S19. Quantitative analysis of Calnexin-TOMM20 colocalization via immunofluorescence. (ns, no significance; *$p < 0.05$; ***$p < 0.001$; mean ± SE, n = 3)

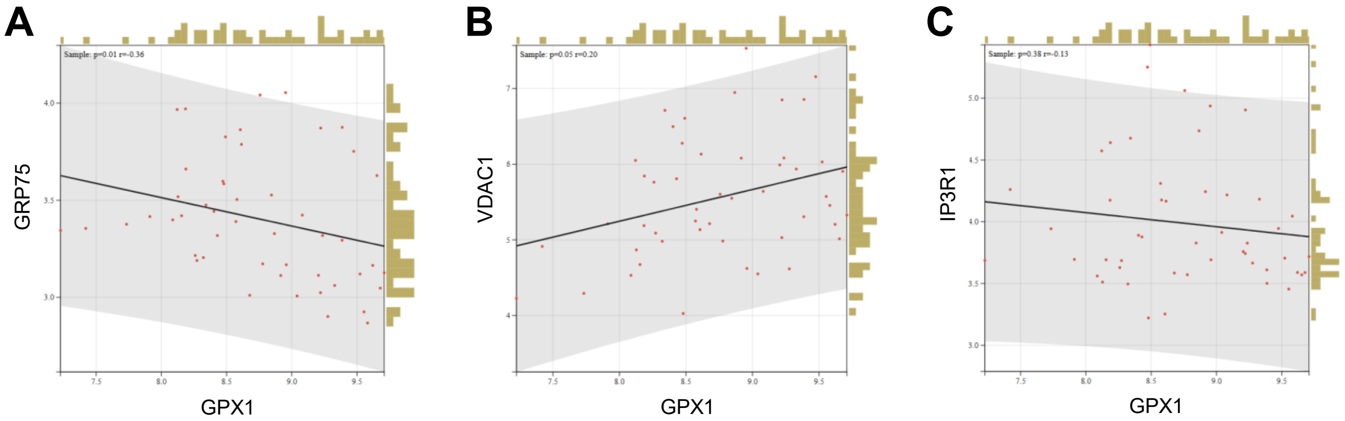

Figure S20. Correlation validation of MAMs-related gene expression in clinical tissues. (A) Spearman correlation between GPX1 and GRP75. (B) Correlation between GPX1 and VDAC1. (C) Correlation between GPX1 and IP3R1.

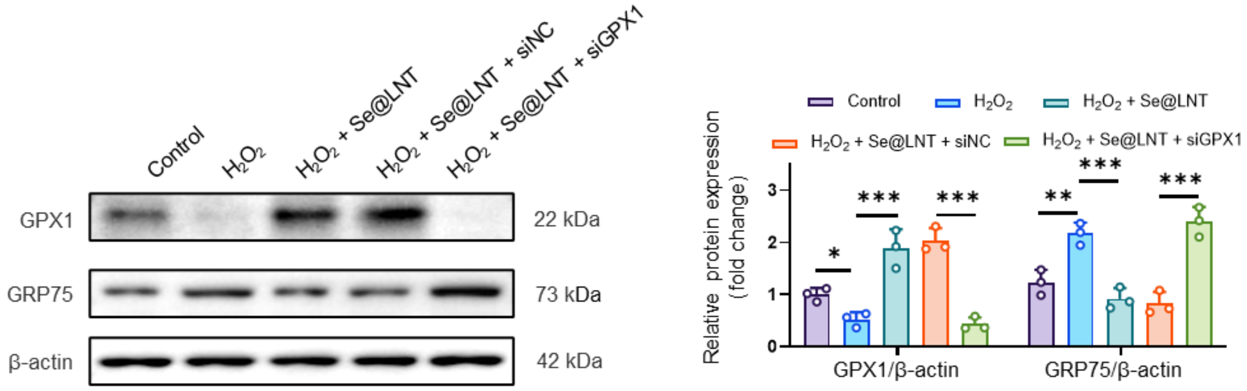

Figure S21. Western blot analysis and quantification of GRP75 expression following GPX1 knockdown. (*$p < 0.05$; **$p < 0.01$; ***$p < 0.001$; mean ± SE, $n = 3$)

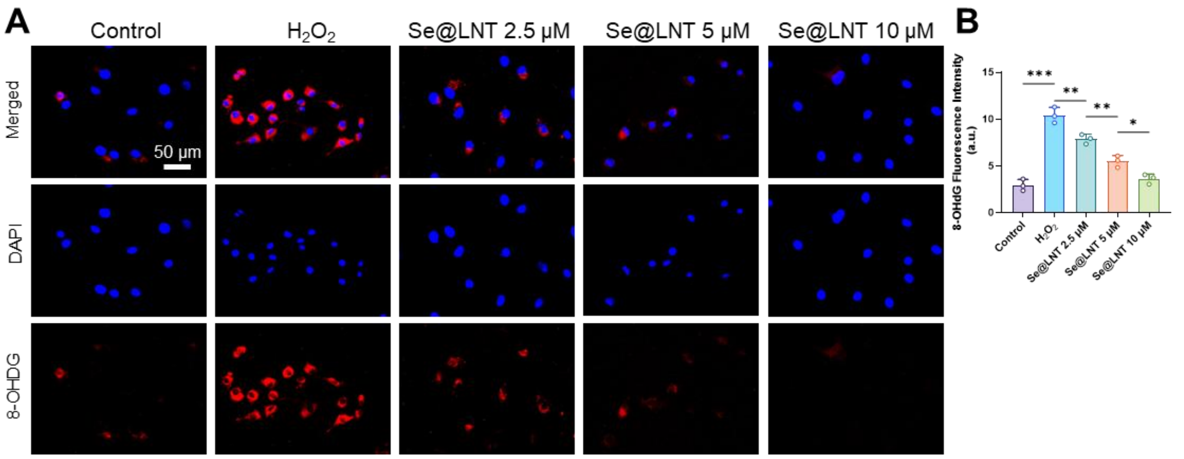

Figure S22. Detection of 8-OHDG levels before and after Se@LNT treatment. (A) Representative fluorescence staining. (B) Quantitative analysis. Scale bar: 50 μm. (*$p < 0.05$; **$p < 0.01$; ***$p < 0.001$; mean ± SE, n = 3)

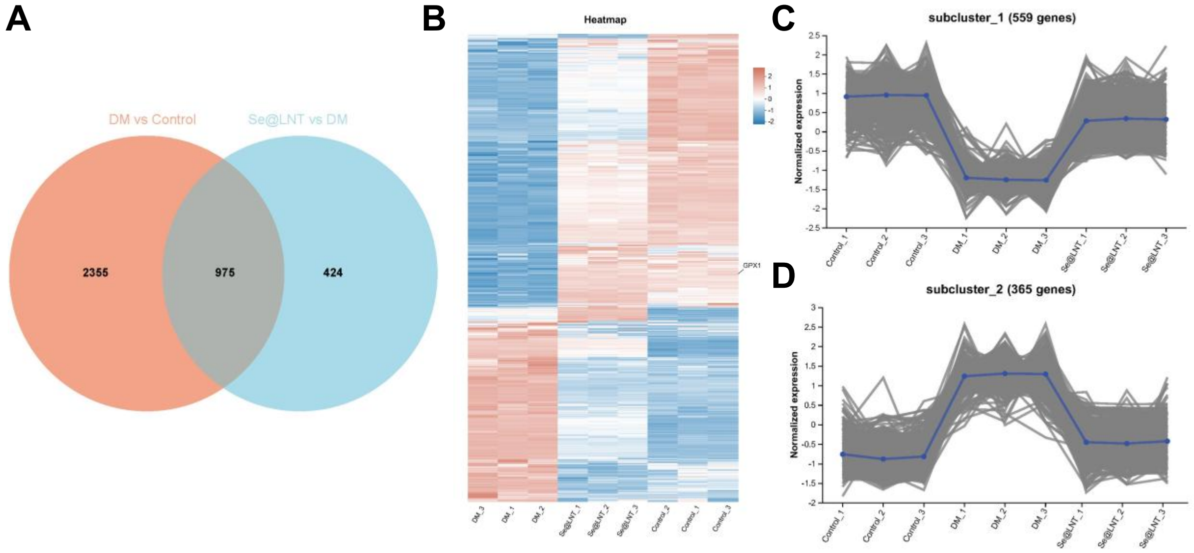

Figure S23. Transcriptomic validation of selenium nanoparticle regulatory networks. (A) Venn diagram analysis of differentially expressed genes between the DM vs. Control and Se@LNT vs. DM groups (975 overlapping genes). (B) Volcano plot illustrating global gene expression patterns across the three groups. (C) Expression trends of subcluster_1 genes (n = 559). (D) Expression trends of subcluster_2 genes (n = 365).

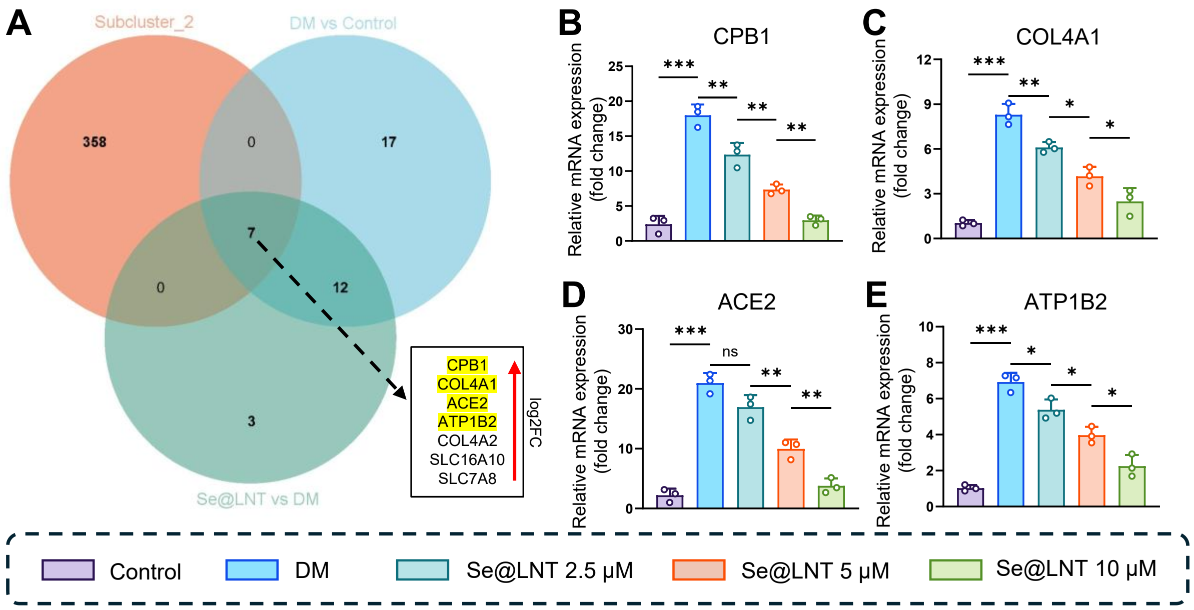

Figure S24. qPCR validation of ER stress-related genes enriched in the "Protein digestion and absorption" pathway. (A) Venn diagram showing the overlap among DEGs from DM vs. Control (36 genes), Se@LNT vs. DM (22 genes), and subcluster_2 (365 genes), identifying seven shared genes. (B-E) qPCR analysis of CPB1, COL4A1, ACE2, and ATP1B2 expression in Control, DM, and Se@LNT-treated groups. (ns, no significance; **p* < 0.05; ***p* < 0.01; ****p* < 0.001; mean ± SE, *n* = 3)

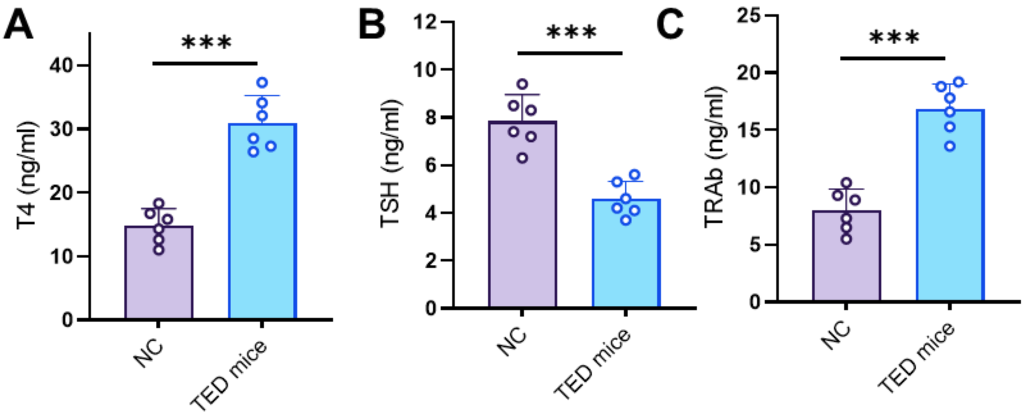

Figure S25. Serum thyroid-related parameter validation in the TED mouse model. (A) Serum thyroxine (T4) levels measured by ELISA. (B) Serum thyroid-stimulating hormone (TSH) levels. (C) Serum TSH receptor antibody (TRAb) levels. (****p* < 0.001; mean ± SE, n = 6)

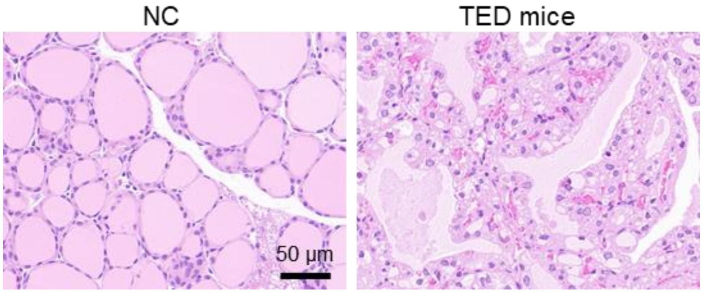

Figure S26. Comparative histopathological analysis of thyroid tissues via H&E staining between the NC and TED mice. Scale bar: 50 μm.

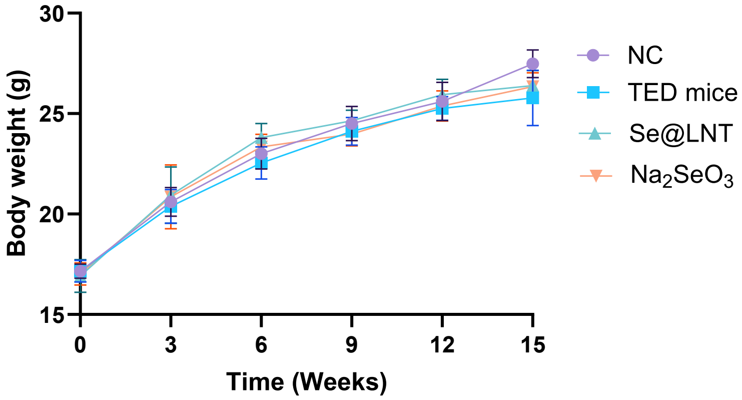

Figure S27. Temporal changes in body weight over 15 weeks among four experimental mouse groups. (mean ± SE, n = 5)

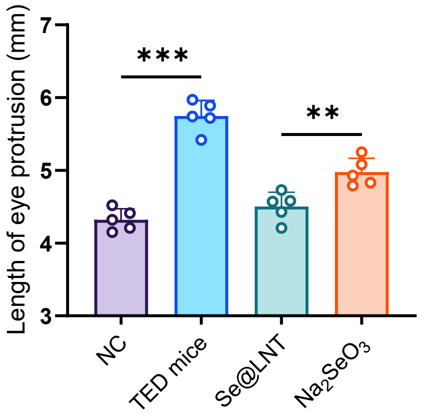

Figure S28. Exophthalmos measurements in four experimental mouse groups. (**p < 0.01; ***p < 0.001; mean ± SE, n = 5)

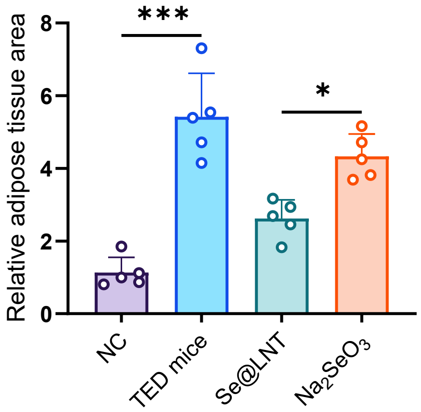

Figure S29. Quantitative analysis of Oil Red O staining in orbital adipose tissues. (*$p < 0.05$; ***$p < 0.001$; $\bar{x} \pm$ se, n = 5)

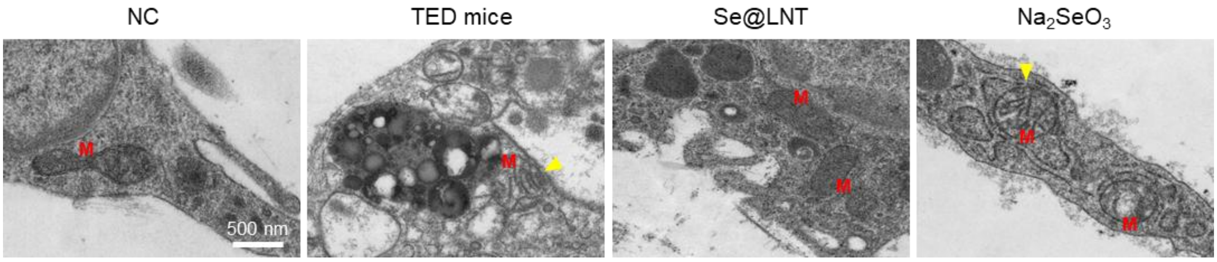

Figure S30. Representative TEM images of orbital fibroblast-rich tissues from each group. Mitochondria are indicated by red "M" labels, and mitochondria-associated membranes (MAMs) are marked with yellow triangles. (Scale bar: 500 nm)

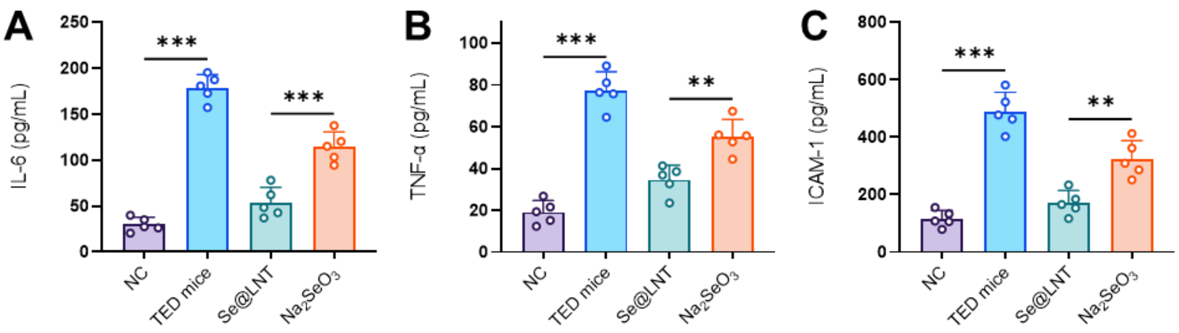

Figure S31. Serum inflammatory cytokine levels in TED mice after selenium treatment. (A) IL-6, (B) TNF-α, and (C) ICAM-1 levels in mouse serum were measured by ELISA. (**$p < 0.01$; ***$p < 0.001$; mean ± SE, n = 5)

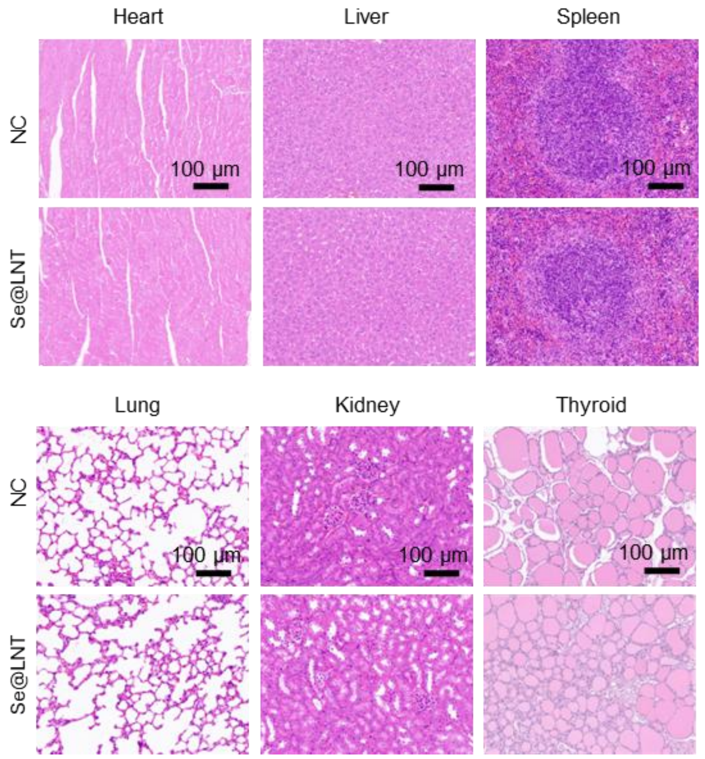

Figure S32. HE staining of major organs in control and Se@LNT-treated mice. Scale bar: 100 μm.

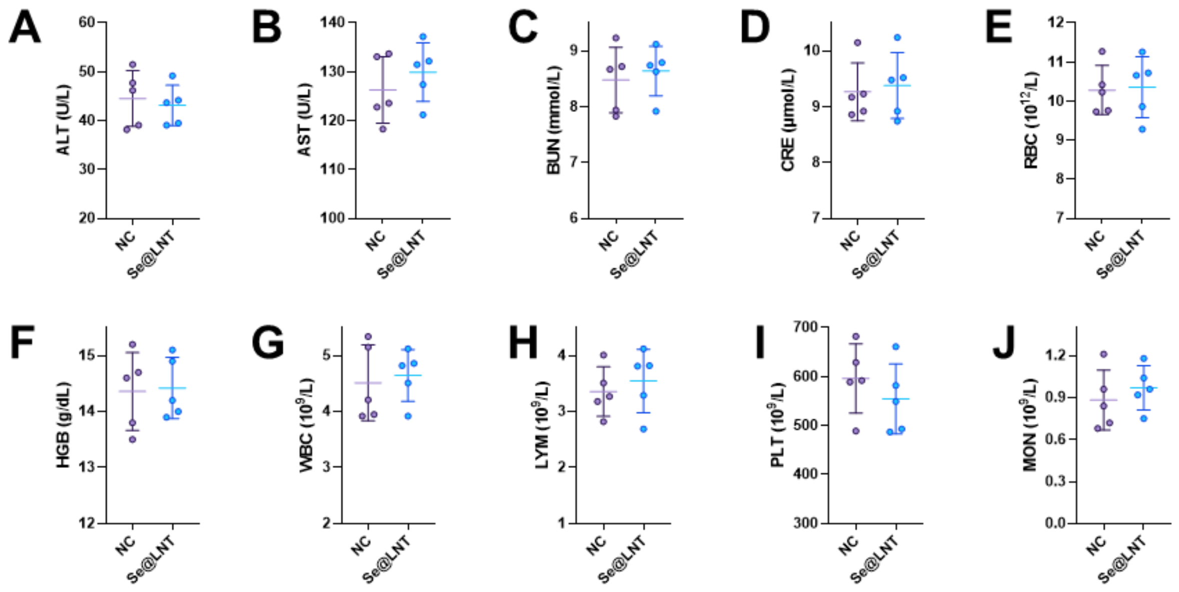

Figure S33. Biochemical and hematological indices in control and Se@LNT-treated mice. ($\bar{x}$ ± se, n = 5)

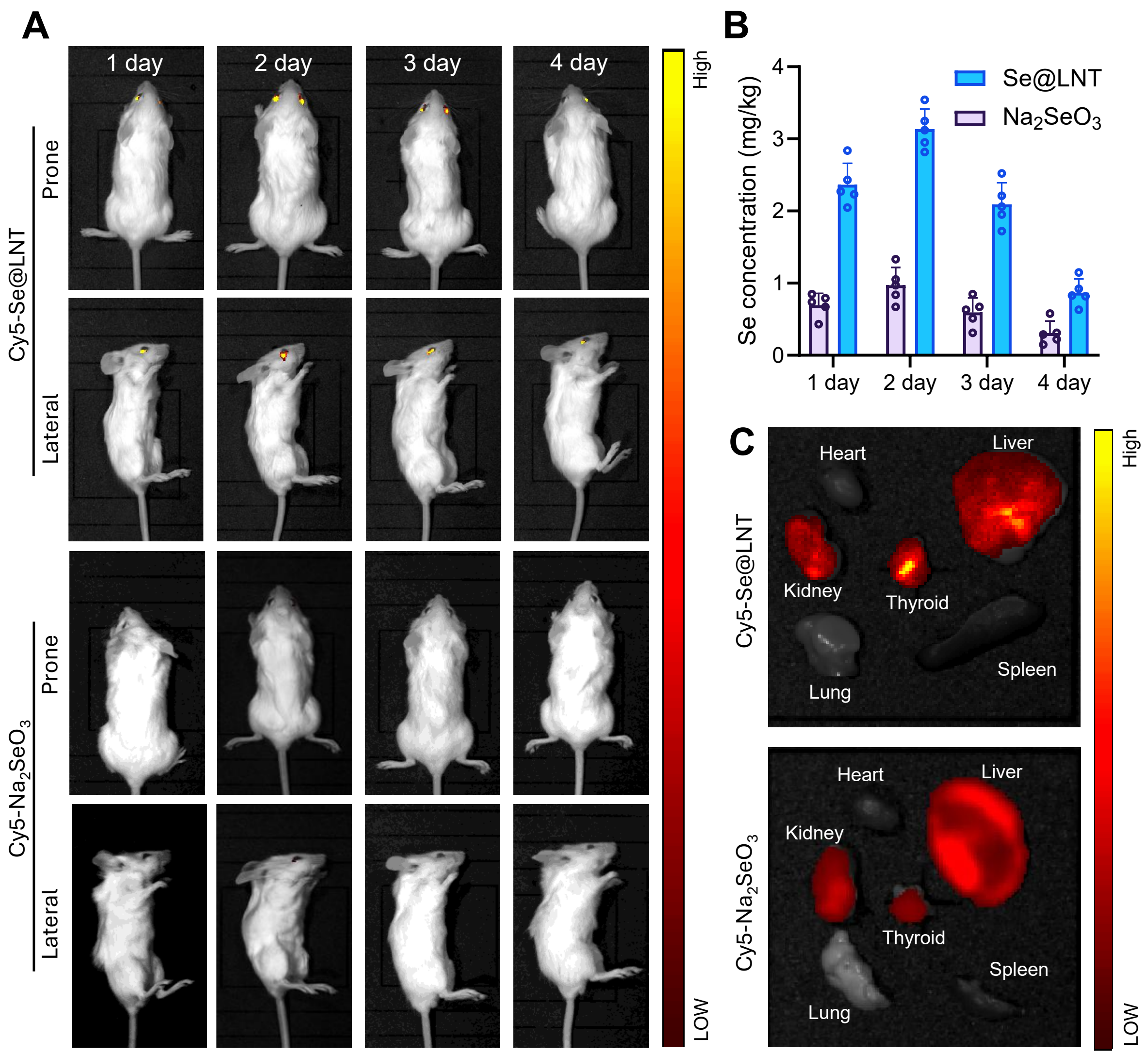

Figure S34. *In vivo* targeting and selenium accumulation analysis in mice. (A) Fluorescence imaging of orbital tissues acquired over 1-4 days using an *in vivo* fluorescence imaging system. Upper panel: prone position; lower panel: lateral position. (B) Selenium content in orbital tissues at indicated time points quantified by ICP-MS ($\bar{x} \pm$ se, n = 5). (C) Fluorescence imaging of major organs 3 days post-injection using the *in vivo* fluorescence imaging system.

Table S1. Clinical Characteristics of Enrolled Subjects.

Parameter	TED (n = 6)	Control (n = 6)
Gender (male/female)	2 / 4	3 / 3
Age (years)	43.6 ± 16.7	39.7 ± 16.2
Clinical Activity Score[a)]	1.7 ± 1.3	\

Parameter	TED (n = 6)	Control (n = 6)
TED Duration (months)	8.3 ± 6.5	\
**Thyroid Function at Enrollment**		
Euthyroid	5	6
Hyperthyroid	1	0
Hypothyroid	0	0
**Treatment History**		
Systemic steroids (within 6 months)	3	0
Radioactive iodine therapy	1	0
Thyroidectomy	0	0
Antithyroid therapy	2	0
**Smoking Status**		
Current smoker	3	3
Former smoker	1	0
Non-smoker	2	3

[a)] The Clinical Activity Score (CAS) was assessed on a 0-7 scale according to the 2021 European Group on Graves' Orbitopathy (EUGOGO) Clinical Practice Guidelines, evaluating seven parameters: eyelid swelling, eyelid erythema, conjunctival redness, chemosis, caruncular edema, spontaneous orbital pain, and gaze-evoked orbital pain [5].

TED: Thyroid Eye Disease; GD: Graves' Disease.

Table S2. Primer sequences for qPCR detection.

Genes	Forward Sequence	Reverse Sequence
DIO1	GGACACCATGCAGAACCAGA	CCTCCCGTTGGTCACCTAGA
DIO2	GGAAGATCGATGTGCAGCAG	GCCGGACTTCTTGAAGGTTG
DIO3	CAGCACATCCTCGACTACGC	TGCTGTGGGATGATGTAGGG

GPX1	ACATCGAGCCTGACATCGAA	TCTGGCAGAGACTGGGATCA
GPX2	GGGACTTCACCCAGCTCAAC	GGTAGGCGAAGACAGGATGC
GPX3	CAACGTGGCCAGCTACTGAG	GACAAAGCCTCCACCTGGTC
GPX4	AGTTTTCCGCCAAGGACATC	TGCTTCCCGAACTGGTTACA
GPX5	GGCAAGCACATCCTCTTCGTCA	GTTATCTCCTGGTTCTTGCTTTCC
GPX6	GCTTTGTCCCCAGTTTCCAG	TGCATGACAGGGACTCCATC
GPX7	CGACTTCAAGGCGGTCAACATC	TCGGTAGTGCTGGTCTGTGAAG
GPX8	CTAGGATCTGAAGGAGAACCTGC	GCCTGATGACTTCAATGGGCTC
TXNRD1	GGGTCCAAATGCTGGAGAAG	GCAGTCTTGGCAACAGCATC
TXNRD2	CCCCGACACTCAGAAGATCC	ATACTCCAGCGGGGTGAAGA
TXNRD3	TCTGGCCTCTTGAATGGACA	GGGGTGAATTCCAATGGTGT
SELENOF	CCTGATTGCAGAGGATGCTG	AGGGTCTGAACCACGGACAT
SELENOH	CCGCTGTAGGAGCAGAGCTT	GCCAGCTTCTCTCGCTTCTC
SELENOI	TGGGAGTTGAGGCCTGGTAT	GACCAAAGGATCCACGCTGT
SELENOK	AAGTGTTGGACAGCCGGAGT	CTAGGGCCACGCAGATGATT
SELENOM	TGACAGCTGAACCGCCTAAA	TCCTGCACTAGCGCATTGAT
SELENON	AGCCAAGGCTGAGAACAAGC	GGGACGAGTTCTCCTGGTTG
SELENOO	ACGGTTGTGTTGCGTGTAGC	GCATTTCTCTGCACGCTGTC
SELENOP	CTCCTCCAGGCCTTCATCAC	GACAATGGCAGCATCAGCTC
SELENOS	AAGAAGCCCCAGGAGGAAGA	CCCTTGGTCAAGAAGCAACC
SELENOT	AGGCGGGTGTTTGAGGAGTA	TATTTTCTTGGCCCCACTGC
SELENOW	GTCGTTTATTGTGGCGCTTG	CGTAGCCATCGCCTTTCTTC
FAPB4	AGTCAAGAGCACCATAACC	GAACTTCAGTCCAGGTCAA
CEBPA	CACGAAGCACGATCAGTC	TGGCAAGTATCCGAGCAA
PLIN1	CTCTGATTCTATGGCTTGGT	GAGTGGTGACAGGAGTTAC
PPARG	TGTCTCATAATGCCATCAGG	TCAGCGGACTCTGGATTC
CPB1	AGACTCGCTCCACCCATACT	CGGCAGGTCCACAGTAAGTT
COL4A1	CCATTTCCGTGGTTTCTCAT	AGGCGACGAAAGAGGAAGA
ACE2	CAAGAGCAAACGGTTGAACAC	CCAGAGCCTCTCATTGTAGTCT
ATP1B2	CTCATGTACTTCCCCTACTATG	ATGCGACATTCTACATTCAC
β-actin	AACTACCTTCAACTCCATCA	GTGTAACGCAACTAAGTCAT
MTND1	CTCTTCGTCTGATCCGTCCT	TGAGGTTGCGGTCTGTTAGT

MTND2	GTAGACAGTCCCACCCTCAC	TTGATCCCGTTTCGTGCAAG
gDNA	GTAACCCGTTGAACCCCATT	CCATCCAATCGGTAGTAGCG

References

1. Zou B, Xiong Z, Yu Y, Shi S, Li X, Chen T. **Rapid selenoprotein activation by selenium nanoparticles to suppresses osteoclastogenesis and pathological bone loss**. *Adv Mater*. 2024; **36**: 2401620.
2. Rosenbaum JT, Choi D, Wilson DJ, Grossniklaus HE, Harrington CA, Dailey RA. *et al.* **Fibrosis, gene expression and orbital inflammatory disease**. *Br J Ophthalmol*. 2015; **99**: 1424-9.
3. Yan J, Zheng Y, Min Z, Ning Q, Lu S. **Selenium effect on selenoprotein transcriptome in chondrocytes**. *Biometals*. 2013; **26**: 285-96.
4. Wang W, Zhang J-W, Qin Y-J, Li H-Y, Dai Y-X, Li H. **Establishment and comparison of two different animal models of graves' orbitopathy**. *Transl Vis Sci Technol*. 2023; **12**: 12-.
5. Bartalena L, Kahaly GJ, Baldeschi L, Dayan CM, Eckstein A, Marcocci C. *et al.* **The 2021 european group on graves' orbitopathy (eugogo) clinical practice guidelines for the medical management of graves' orbitopathy**. *Eur J Endocrinol*. 2021; **185**: G43-G67.
